# Income, education, and other poverty-related variables: A journey through Bayesian hierarchical models

**DOI:** 10.1016/j.heliyon.2024.e27968

**Published:** 2024-03-15

**Authors:** Irving Gómez-Méndez, Chainarong Amornbunchornvej

**Affiliations:** National Electronics and Computer Technology Center (NECTEC), Thailand

## Abstract

One-shirt-size policy cannot handle poverty issues well since each area has its unique challenges, while having a custom-made policy for each area separately is unrealistic due to limitation of resources as well as having issues of ignoring dependencies of characteristics between different areas. In this work, we propose to use Bayesian hierarchical models which can potentially explain the data regarding income and other poverty-related variables in the multi-resolution governing structural data of Thailand. We discuss the journey of how we design each model from simple to more complex ones, estimate their performance in terms of variable explanation and complexity, discuss models' drawbacks, as well as propose the solutions to fix issues in the lens of Bayesian hierarchical models in order to get insight from data.

We found that Bayesian hierarchical models performed better than both complete pooling (single policy) and no pooling models (custom-made policy). Additionally, by adding the year-of-education variable, the hierarchical model enriches its performance of variable explanation. We found that having a higher education level increases significantly the households' income for all the regions in Thailand. The impact of the region in the households' income is almost vanished when education level or years of education are considered. Therefore, education might have a mediation role between regions and the income. Our work can serve as a guideline for other countries that require the Bayesian hierarchical approach to model their variables and get insight from data.

## Highlights

• Hierarchical models can explain income and poverty-related variables for the regions of Thailand.
• Having a higher education level increases significantly the households' income for all the regions in Thailand.
• Education might have a mediation role between regions and income.
• For each year-of-education the monthly income is increased in 1880 THB on average, vary between 1278 THB up to 2545 THB.
• On average, monthly income with high education is 8325 THB higher than mid education and 14844 THB higher than low education.

## 1 Introduction

### 1.1 Related works

Before solving poverty and inequality issues, one needs a tool to measure the degree of how severe the issues in a given area are. In the past, poverty was about lacking of income. However, the concept of poverty is complex and multidimensional [1], [2], which implies that solving only monetary problems is not enough to alleviate the issues of poverty.

Therefore, the Multidimensional Poverty index (MPI) in [3], [4] was developed and used by United Nations Development Programme (UNDP) as a standard way to measure poverty in multidimensions such as living standard, health, education, etc. The MPI indices from nations around the world have been reported annually by UNDP. In the aspect of inequality, the well-known index that is typically used for measuring income inequality is the Gini coefficient [5]. The index represents the distribution of resources among people. In [5], the authors used the Gini index to measure income inequality in Sub-Saharan Africa. The work in [6] used Gini index as an income inequality measure to find association between a number of COVID19 cases and income inequality in USA. Instead of the Gini coefficient, the work in [7] proposed the use of the network density of income gaps (edges represent significant gaps) to measure income inequality among different occupations.

Even though these indices provide rich information regarding poverty and income inequality in each area, they never provide the information of resolution of poverty issues; given multiple areas, it is impossible to tell from MPI whether these areas share similar issues and need only a single policy to solve poverty. To address this gap, the work in [8] uses both minimum description length (MDL) and Gaussian Mixture Models [9], [10], [11] to find optimal multiresolution partitions that can place a single policy for each partition since each one represents an area that have a similar model of issues. However, these works cannot be used to provide insights regarding dependencies of issues between different area resolution levels. Does income variable in the national level affects income variables in provinces or lower levels? The next section provides the reasons of using Bayesian hierarchical models in our work.

### 1.2 Relevance of Bayesian hierarchical models

One of the approaches to model policies is to use Bayesian's statistics and modeling, which is widely used to model public policies in government setting [12], [13] as well as public opinions [14], [15]. Specifically, for example, in [13], Bayesian approaches were used to evaluate public programs, while, in [15], Bayesian approaches were used to evaluate internet public opinions.

In this work, we propose to use Bayesian hierarchical models to analyze variables that are related to poverty and inequality from a population dataset of Thai households.

Some datasets are collected with an inherent multilevel structure, for example, households within a region of a country. Then, hierarchical modeling is a direct way to include clusters at all levels of a phenomenon, without being overwhelmed with the problems of overfitting. At a practical level, hierarchical models are flexible tools combining partial pooling of inferences. They have been successfully involved in various practical problems, including biomedicine [16], [17], genetics [18], [19], ecology [20], [21], psychology [22], among others. We refer to [23] for a review on Bayesian modeling and a further list of their applications, including Bayesian hierarchical models.

The traditional alternatives to hierarchical modeling are complete pooling, in which differences between groups are ignored, and no pooling, in which data from different sources are analyzed separately. As we shall discuss, both these approaches present problems at a conceptual level and to explain some data. However, the extreme alternatives can be useful as preliminary estimates.

### 1.3 Motivation

Poverty is one of the most important issues that mankind faces [1]. It is one of the main root causes that harms several aspects of society such as economy development [24], education [25], healthcare systems [26], etc. For each year, there were millions of human deaths causing by poverty [27], [1]. Especially, when the COVID-19 pandemic occurred, deprived people were the ones who suffered the most in servery aspects including casualty [28], [6]. To combat poverty issues, a government needs appropriate policies to solve them [29], [30]. With the proper policies and sufficient resources, poverty can be alleviated effectively. However, finding the right policy is a non-trivial task due to the complexity of issues and unique characteristics of regions. For instance, in a similar problem, one solution in a specific region might not be able to solve it in another region even though they have similar characteristics in many aspects [31], [32].

One-shirt-size policy is a popular way to solve an issue by policy makers since it is simple to implement and typically uses less resources than a custom-made policy that is designed for a specific region. Nevertheless, one-shirt-size policy is unable to handle issues in all regions of a country effectively since each region might have their own unique socioeconomic context or other issues of poverty [33], [34], [35], [8]. On the other hand, making a specific policy for each region to solve their unique problems is impossible due to the limitation of time and resources [8].

### 1.4 Contribution

To find an optimal solution between the two extremes of one-shirt-size and custom-made policies, in this work, we propose to use *Bayesian hierarchical models* [36], [37] to find a proper model that effectively explains target variables (e.g. income, debt, savings, etc.) related to poverty. To the best of our knowledge, there is no work in the literature that makes use of Bayesian hierarchical models to analyze variables of poverty in Thailand. For some variables, we found that complete pooling (representing one-shirt-size policy) and no pooling (representing custom-made policy) cannot explain a target variable while hierarchical models can, which represents the middle ground between these two extremes. Moreover, since Bayesian hierarchical models are able to extract dependencies of issues between different area resolution levels, the models can provide the insight regarding making a policy that takes into account the common ground and uniqueness between different governing layers (e.g. a group of village shares the same issues of education but each village has its unique income issues.) The analyses and results of our work can be used as a role model for the analysis in other countries.

The rest of the article is organized as follows. In Section 2 we explain our methodology to select the variables studied in this work, how simple and complex models interact between them, and the criterion used to compare different models. In Section 3 we introduce the hierarchical model as a trade-off between no pooling and complete pooling models. In Section 4 we present a hierarchical model that incorporates two non-nested clusters. Section 5 is devoted to Bayesian hierarchical regression. In Section 6 we discuss the principal results observed throughout this work. Finally, Section 7 presents the conclusions.

## 2 Methodology

With the proliferation of Bayesian methods (see [23] for a list of open Bayesian software programs), they have become easier to build and implement than to understand what they are doing. In an attempt to narrow this gap, in this work we present a comprehensive framework for hierarchical models. Thus, we do not only show how to implement these models, but also how to interpret the parameters according to the level where they belong in the hierarchy and their relation with other parameters.

Instead of starting directly with the hierarchical models, we begin with the extreme cases of no pooling and complete pooling. It is only after analyzing their implications and their lack to explain adequately certain aspects of the data, that we introduce the hierarchical models as a way to mitigate these problems. Thus, every time we introduce a new hierarchical model is always as an extension of a previous one.

Noninformative priors (also known as reference priors or objective priors) are notoriously difficult to derive for many hierarchical models. Thus, throughout this work, we present an approach in which simpler models are used for prior specification in more complex models. This contrasts with the most common approach to prior specification in which a prior distribution is selected because it has been previously used in the literature. Based on the assumption that the community of people using that prior is doing it for a good reason. However, as pointed out by [38], most of these priors have been chosen for specific problems and might be inappropriate for others. Furthermore, as commented by [39]:

There's an illusion sometimes that default procedures are more objective than procedures that require user choice, such as choosing priors. If that's true, then all “objective” means are that everyone does the same thing. It carries no guarantees of realism or accuracy.

As commented by [40], hierarchical models allow a more “objective” approach to inference by estimating the parameters of prior distributions from data rather than requiring them to be specified using subjective information. Moreover, in hierarchical models where priors depend on hyperparameter values that are data-driven avoids the direct problems linked to double-dipping [23]. Therefore, our approach follows the tendency by part of the Bayesian community to move from noninformative priors [36], [39], [41], [42]. We do not claim that the proposed approach is optimal. Instead, we make the more modest claim that it is useful for practical purposes.

To show the potential of our approach, in Appendix C we present a brief simulation study. However, it is important to emphasize that performing a simulation study should not be treated as a simple task that can be incorporated easily. There are just too many variables, models and factors without a trivial answer: what is the distribution to be used for simulating the data? which aspects of the model are we interested to study? are we interested in the robustness of the methods when outliers are presented? their computational efficiency? or their behavior when the assumed distribution in the model does not correspond with the distribution of the data? how many observations should be simulated for the training phase? how many for the testing phase? the distribution for both phases should be maintained the same? Many decisions should be made for an appropriate simulation study. Thus, a complete simulation analysis is out of the scope and aim of this article. For seek of brevity the simulation studies explore the Bayesian hierarchical models with one cluster and the Bayesin hierarchical regression models.

### 2.1 Data and related information

The dataset used throughout this work has been collected in 2022 by Thai government agencies. The main purpose of this dataset is for supporting government policy makers to calculate MPI to support poverty alleviation policy making in the Thai People Map and Analytics Platform project (www.TPMAP.in.th) [8]. The original dataset has 12,983,145 observations (each one corresponding to a household). However, there were 569 households that declared having no-income, which represents 0.0044% of the observations. We consider this percentage negligible and do not consider them for further analyses. The number of households consulted for each province goes from 44,012 up to 645,433, then, for all practical purposes, we can ignore the uncertainty within each province.

### 2.2 Comparison of models

Since different models are implemented for the same data and variables, we need to develop a methodology that allows us to compare them. For this purpose, we considered the Widely Applicable Information Criterion (WAIC) [43], renamed in [44] as the Watanabe-Akaike Information Criterion. This information criterion allows a fair comparison between models of different complexity. Compared to other information criteria like the Akaike Information Criterion (AIC) [45], WAIC averages over the posterior distribution rather than conditioning on a point estimate (like the maximum likelihood estimator), making it a more suitable criterion for Bayesian models. Moreover, AIC is defined relative to the maximum likelihood estimate and so is inappropriate for hierarchical models.

It is important to point out that many non-Bayesian models are equivalent to Bayesian models, usually when noninformative vague priors are considered, and thus for such models the WAIC can also be calculated, which allows a direct comparison between non-Bayesian and Bayesian models. However, other strategies can be considered as well, such as calculating a loss function, as could be the negative of the log-likelihood, over a testing data set or through cross-validation. Unfortunately, some of these techniques like cross-validation might required a lot of computational resources, especially in the extreme case of leaving-one-out cross validation. Thus, since it is not restrictive computationally and for its suitability for Bayesian hierarchical models, in this article we focus our attention into the WAIC for the comparison of models

We emphasize that the WAIC is just another statistical summary of our models, and it is not, in any way, a substitute of an appropriate analysis of the models and their results. If we find that the model does not fit for its intended purposes, we are obliged to search for a new model that fits. Then, understanding different aspects of the models and their implications must be the principal guide for their selection and comparison. See [46] for further discussion on Bayesian predictive model assessment, selection, and comparison methods. In Appendix A.1 we provide further details about the WAIC.

### 2.3 Variables to analyze

For its direct relation with poverty, we exemplify our methodology throughout this work considering the monthly average income in households. However, this approach is suitable to be applied to a widely variety of variables. To decide the variables where to apply our methodology, we first calculated the percentage of households in each province affected by the variables considered in the dataset. In Fig. 1, we present boxplots for the 10 issues with the largest percentage.

**Figure 1:**
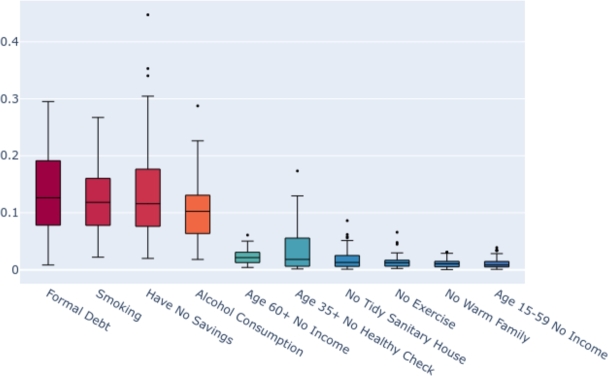
Boxplots of the 10 poverty-related variables affecting the largest percentage of households in Thailand.

Due to the large percentage of households affected by (having) formal debt, smoking, having no savings, and alcohol consumption, as well as their large variance between provinces, we also analyzed these variables with the different models presented in this work.

## 3 Hierarchical model with one cluster: income per region

### 3.1 No pooling model and complete pooling model

#### 3.1.1 No pooling model

Let $Y_{ij}$ be the average household in the province *i*, which belongs to the region *j*, each region with $n_{j}$ observations. We consider 6 regions for Thailand: Northern Thailand, Northeast Thailand, East Thailand, Central Thailand, Western Thailand and Southern Thailand.

Before jumping directly into our hierarchical model, we first consider separate independent models for each region. Thus, each region has its own mean $\theta_{j}$ and its own variance $\sigma_{j}^{2}$. We assume that $Y_{ij}|\theta_{j},\sigma_{j}^{2}∼N(\theta_{j},\sigma_{j}^{2})$,^1^ j=1,…,J. This model is represented graphically on the left of Fig. 2. For a simple model like this, we can consider vague noninformative priors without any harm, thus we use the well-known noninformative prior^2^
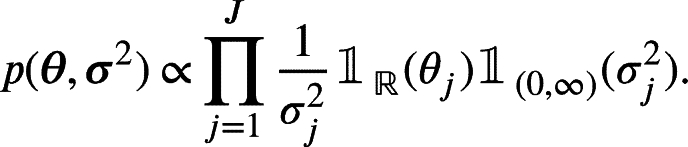

**Figure 2:**
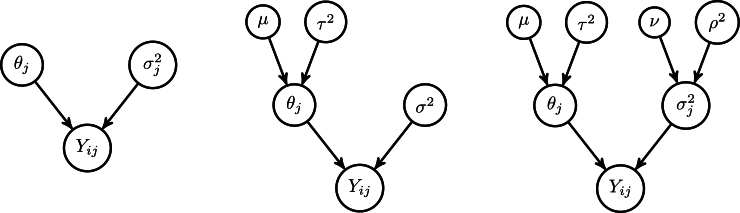
Graphical representation of the considered models. Left: Separate independent models for each region, if we add the restrictions *θ*_*j*_ = *θ* and $\sigma_{j}^{2}=\sigma^{2}$ for all *j*, then we get the complete pooling model. Center: Hierarchical model, where the regional means share a common structure, yet allowing them to be different, but the constraint of equal variance is still present. Right: Hierarchical model imposing a common structure for both, the regional means and the within-region variances.

It is not difficult to prove that the conditional posterior distributions for each $\theta_{j}$ and $\sigma_{j}^{2}$ are given by^3^

$$\theta_{j}|\sigma^{2},Y∼N({\overset{¯}{Y}}_{\cdot j},{\overset{¯}{\sigma }}_{j}^{2}),\sigma_{j}^{2}|\theta ,Y∼\text{Inverse-}\chi^{2}(n_{j},v_{j}),$$

where

$${\overset{¯}{Y}}_{\cdot j}=\frac{1}{n_{j}}\sum_{i=1}^{n_{j}}Y_{ij},\;{\overset{¯}{\sigma }}_{j}^{2}=\frac{\sigma_{j}^{2}}{n_{j}},\;\text{and }v_{j}=\frac{1}{n_{j}}\sum_{i=1}^{n_{j}}{\left(Y_{ij}-\theta_{j}\right)}^{2}.$$

Which makes it straightforward to simulate from the joint posterior distribution using Gibbs sampling [47].

On the top left of Fig. 3, we present the estimated mean for each region, $\theta_{j}$, with a credible interval of 0.95 posterior probability. We observe that, since we considered noninformative priors, the estimations are centered on the observed regional averages. Note also that, because the regions share no information between them, the credible intervals are large, especially for regions with few provinces. On the top right, we present a similar plot for the standard deviation for each region, $\sigma_{j}$. On the bottom, we present credible intervals for the average monthly income in each region. We present these credible intervals with the observed average for the provinces belonging to the region.

**Figure 3:**
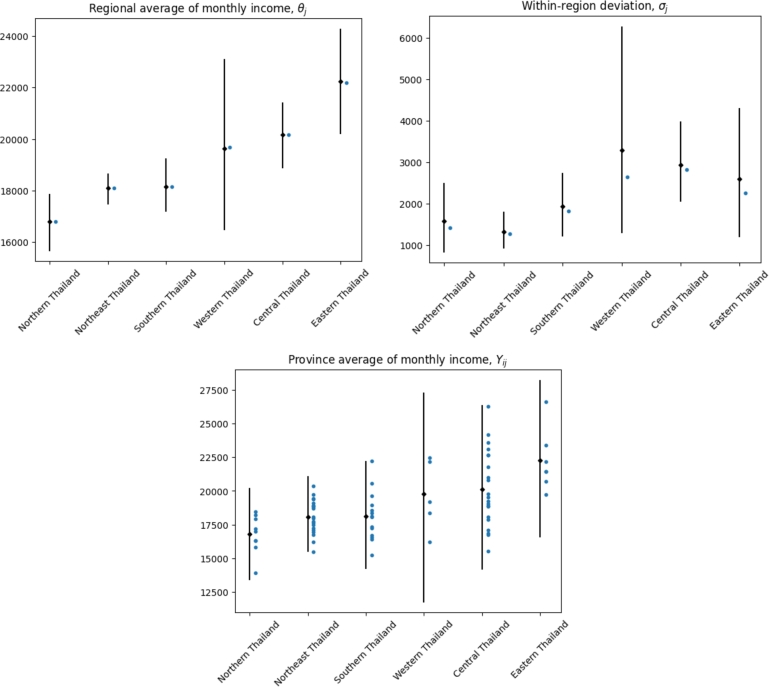
Results considering independent separate models for each region. On the top row we show the regional mean, *θ*_*j*_ (left) and the regional standard deviation, *σ*_*j*_ (right). On the bottom we show the province mean.

#### 3.1.2 Complete pooling model

We can observe in Fig. 3 that the intervals overlap for most of the regions. This overlapping suggests that all the parameters might be estimating the same quantity. In fact, it is highly unlikely that the regions are independent between them, which makes difficult to justify an independent model for each one. Thus, we can consider the complete pooling model, in which all the regional means, $\theta_{j}$, and their variances, $\sigma_{j}^{2}$, are equal to some common values *θ* and $\sigma^{2}$, respectively. That is, for the complete pooling model, we assume that $Y_{ij}|\theta ,\sigma^{2}∼N(\theta ,\sigma^{2})$. Similar to the no pooling model, the complete pooling is represented by the graph on the left of Fig. 2, with the constraints that $\theta_{j}=\theta$ and $\sigma_{j}^{2}=\sigma^{2}$ for all j=1,…,J.

For this model we still consider the noninformative prior distribution for *θ* and $\sigma^{2}$,
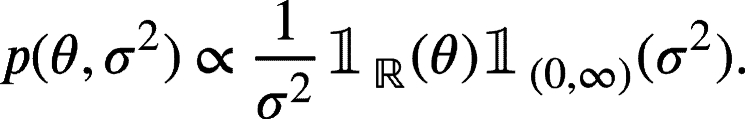
 It is not difficult to prove that the conditional posterior distribution of *θ* is given by $\theta |\sigma^{2},Y∼N({\overset{¯}{Y}}_{\cdot \cdot },\varphi^{2})$, where

$${\overset{¯}{Y}}_{\cdot \cdot }=\frac{\sum_{j=1}^{J}\frac{{\overset{¯}{Y}}_{\cdot j}}{{\overset{¯}{\sigma }}_{j}^{2}}}{\sum_{j=1}^{J}\frac{1}{{\overset{¯}{\sigma }}_{j}^{2}}},\;\varphi^{2}=\frac{1}{\sum_{j=1}^{J}\frac{1}{{\overset{¯}{\sigma }}_{j}^{2}}},\;\text{and }{\overset{¯}{\sigma }}_{j}^{2}=\frac{\sigma^{2}}{n_{j}},$$

while the conditional posterior distribution of $\sigma^{2}$ is given by $\sigma^{2}|\theta ,Y∼\text{Inverse-}\chi^{2}(n,{\overset{ˆ}{\sigma }}^{2})$, with

$$n=\sum_{j=1}^{J}n_{j},\;\text{and }{\overset{ˆ}{\sigma }}^{2}=\frac{1}{n}\sum_{j=1}^{J}\sum_{i=1}^{n_{j}}{\left(Y_{ij}-\theta \right)}^{2}.$$

Once again, having access to the conditional posterior distributions allows us to simulate from the joint posterior distribution using Gibbs sampling.

We present in Fig. 4 the analogous results of Fig. 3 for the complete pooling model. Comparing both Figures, we observe much narrower intervals (also calculated at a 0.95 posterior probability), this is because now we are using all the observations to estimate the same common quantities, reducing the uncertainty significantly. However, we observe that the common mean *θ* can barely explain the mean of a few regions, being an unreliable estimate for the regions with the largest and smallest means. We can also observe that the estimator of the common within-region deviation is not centered around the average of the observed sample deviations, but upward. This is because now that we have constraint the regional means to be all the same, the only way to explain the variation throughout the observations is by estimating a higher value for the common deviation *σ*. Note also, on the bottom row of Fig. 4, that all the provinces in Northern Thailand are on the below half of the credible interval, while all the provinces in Eastern Thailand are above. We can conclude from all these observations that a complete pooling model is inappropriate.

**Figure 4:**
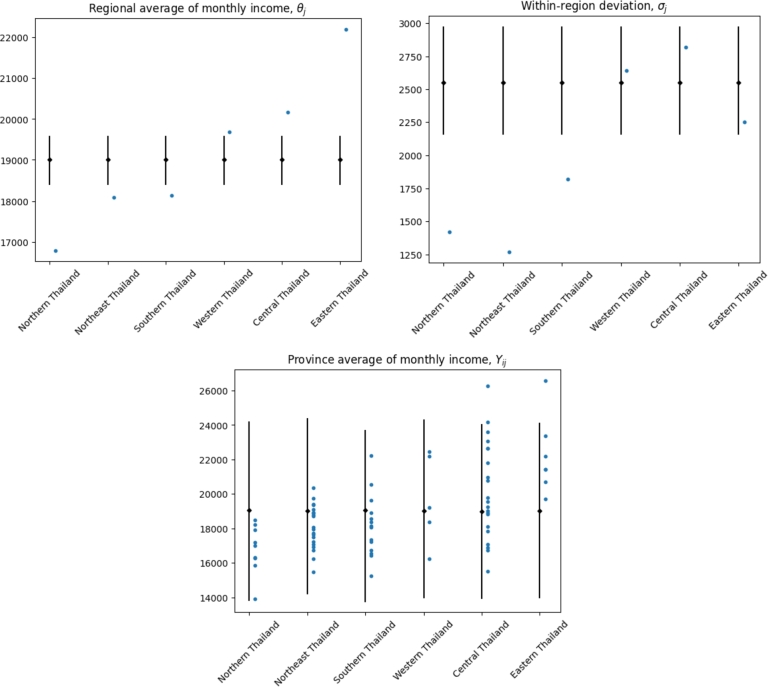
Results considering the complete pooling model for all the regions. On the top row we show the regional mean, *θ*_*j*_ (left) and the regional standard deviation, *σ*_*j*_ (right). On the bottom we show the province mean.

### 3.2 Hierarchical model with common within-cluster variance

Because considering independent models for each region seems difficult to justify and we observe a poor performance for the complete pooling model, we consider as a better approach a model that makes a trade-off between these two extreme cases. A hierarchical model achieves this compromise.

Instead of adding a hierarchical structure to all the parameters, we propose to add it to one parameter first, and consider more complex models only as suggested by the data after analyzing the results of the previous model. For this reason, we maintain a common within-region variance $\sigma^{2}$, but consider different regional means, $\theta_{j}$. However, these means are not independent, instead they share a common structure. From a statistical perspective this means to abandon the noninformative prior for $\theta_{j}$ and consider a distribution that depends on some hyperparameters, as it is represented by the graph in the center of Fig. 2.

For the simplicity of a conjugate model [42], we consider the prior $\theta_{j}|\mu ,\tau^{2}∼N(\mu ,\tau^{2}),\;j=1,\ldots ,J$. In this model, *μ* represents the national average of the monthly income and *τ* represents the between-regions deviation. For the within-regions variance, $\sigma^{2}$ we still consider the noninformative prior
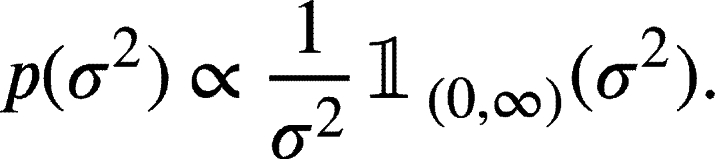


To complete our model, we must assign prior distributions for *μ* and *τ*. However, we must be careful since the usual noninformative distributions for location and scale parameters might lead to the non existence of the posterior distributions. For example, using the usual noninformative prior of a variance parameter for $\tau^{2}$,  yields an improper posterior distribution. Meanwhile, the vague prior
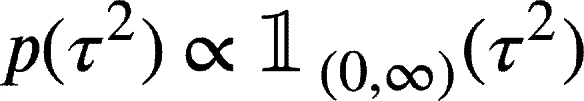
 generates a proper posterior distribution, thus we use this prior for $\tau^{2}$. For *μ*, we use the usual noninformative prior
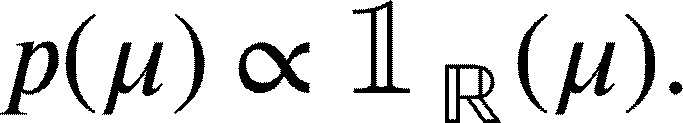


In Appendix A.2, we present an empirical approach (developed in [42]) to estimate these parameters, and explore in more detail the qualitative implications of the priors and the values taken by the parameters in the hierarchical models.

With this model, the following conditional distributions for the parameters can be deduced [42].

*Conditional posterior for*$\theta_{j}$ 

$$\theta_{j}|\mu ,\tau^{2},\sigma^{2},Y∼N({\overset{ˆ}{\theta }}_{j},V_{\theta_{j}}),$$

where

(1) $${\overset{ˆ}{\theta }}_{j}=\frac{\frac{1}{{\overset{¯}{\sigma }}_{j}^{2}}{\overset{¯}{Y}}_{\cdot j}+\frac{1}{\tau^{2}}\mu }{\frac{1}{{\overset{¯}{\sigma }}_{j}^{2}}+\frac{1}{\tau^{2}}},\;\text{and }V_{\theta_{j}}=\frac{1}{\frac{1}{{\overset{¯}{\sigma }}_{j}^{2}}+\frac{1}{\tau^{2}}}.$$

*Conditional posterior for μ* 

$$\mu |\theta ,\tau^{2},\sigma^{2},Y∼N(\overset{ˆ}{\mu },\tau^{2}/J),$$

where $\overset{ˆ}{\mu }=\frac{1}{J}\sum_{j=1}^{J}\theta_{j}$.

*Conditional posterior for*$\sigma^{2}$ 

$$\sigma^{2}|\theta ,\mu ,\tau^{2},Y∼\text{Inverse-}\chi^{2}(n,{\overset{ˆ}{\sigma }}^{2}),$$

where $n=\sum_{j=1}^{J}n_{j}$, and

$${\overset{ˆ}{\sigma }}^{2}=\frac{1}{n}\sum_{j=1}^{J}\sum_{i=1}^{n_{j}}{\left(Y_{ij}-\theta_{j}\right)}^{2}.$$

*Conditional posterior for*$\tau^{2}$ 

$$\tau^{2}|\theta ,\mu ,\sigma^{2},Y∼\text{Inverse-}\chi^{2}(J-1,{\overset{ˆ}{\tau }}^{2}),$$

where

$${\overset{ˆ}{\tau }}^{2}=\frac{1}{J-1}\sum_{j=1}^{J}{\left(\theta_{j}-\mu \right)}^{2}.$$

As we did with the previous two models, we present in Fig. 5 the estimated mean for each region, $\theta_{j}$, the estimated common within-region deviation *σ*, and the average province monthly income, all of them with their respective credible intervals of 0.95 posterior probability. For the regional means, we can observe that the uncertainty is considerable less that when we considered independent analyses (see Fig. 3) without having a poor performance as the complete pooling model. Also, since we accept different means for each region, now the common variance is not overestimated. However, we can observe that a common variance is infeasible to explain the observed variability for most of the regions.

**Figure 5:**
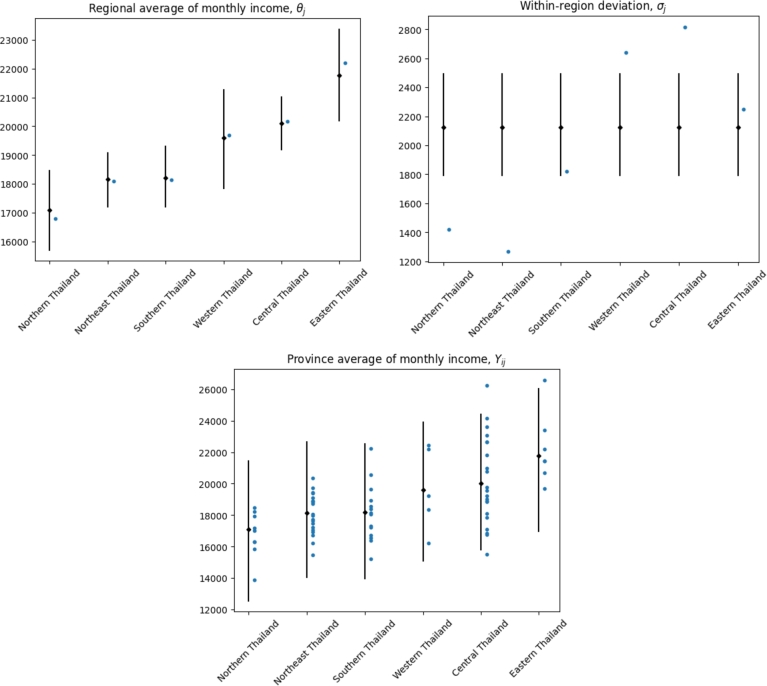
Results considering a hierarchical model. We allow the mean of each region to vary while maintaining the same within-region variance *σ*^2^. On the top row we show the regional mean, *θ*_*j*_ (left) and the regional standard deviation, *σ*_*j*_ (right). On the bottom we show the province mean.

### 3.3 Hierarchical model varying within-cluster variance

Because a common within-region variance $\sigma^{2}$ seems infeasible, we can impose a hierarchical level to it, similarly as we did with the regional means. Thus, we allow each region to have its own variance $\sigma_{j}^{2}$, but all of them sharing a common structure. For simplicity of a conjugate model, we consider the following prior distribution

$$\sigma_{j}^{2}|\nu ,\rho^{2}∼\text{Inverse-}\chi^{2}(\nu ,\rho^{2}).$$

This model is represented graphically on the right of Fig. 2. These graphical representations, called Bayesian networks, meet two objectives. First, they visualize easily the hierarchical relations between variables, which helps with the interpretation of the parameters and the understanding of the model. Second, they allow us to use *d*-separation rules [48] to deduce the conditional independence between the parameters. We suggest [49] and [50] for gentle introductions to Bayesian networks and *d*-separation.

Consider, for example, the graph presented on the right of Fig. 2, while *a priori* $\theta_{j}$ is independent of $\sigma_{j}^{2}$, we can see that conditioning on **Y** creates a dependence between both parameters, that is , but . However, if we condition on both **Y** and $\sigma_{j}^{2}$, $\theta_{j}$ is independent of *ν* and $\rho^{2}$. This implies that the full conditional posterior of $\theta_{j}$ is exactly the same as in the model of Section 3.2, which assumes the same variance for all the regions (with the minor change of defining ${\overset{¯}{\sigma }}_{j}^{2}=\sigma_{j}^{2}/n_{j}$ instead of ${\overset{¯}{\sigma }}_{j}^{2}=\sigma^{2}/n_{j}$). Using the same reasoning, it is easy to see that the posterior distributions of *μ* and $\tau^{2}$ are also the same as those presented in Section 3.2.

Therefore, we only need to calculate the posterior distributions of $\sigma_{j}^{2}$, *ν* and $\rho^{2}$. Due to the conjugacy property of the model, it is not difficult to prove (see [42]) that

$$\sigma_{j}^{2}|\theta ,\nu ,\rho^{2},Y∼\text{Inverse-}\chi^{2}(\nu_{j},{\overset{ˆ}{\sigma }}_{j}^{2}),$$

where

$$\nu_{j}=\nu +n_{j},\;{\overset{ˆ}{\sigma }}_{j}^{2}=\frac{\nu \rho^{2}+n_{j}v_{j}}{\nu +n_{j}},\text{and }v_{j}=\frac{1}{n_{j}}\sum_{i=1}^{n_{j}}{\left(Y_{ij}-\theta_{j}\right)}^{2}.$$

To complete our model, we must assign prior distributions for $\rho^{2}$ and *ν*. Note that, considering the Bayesian network associated to the model, and *d*-separation, it is easy to see that once we condition on $\sigma^{2}$, $\rho^{2}$ is independent of all the other variables and parameters of the model, except for *ν*. For $\rho^{2}$, we can prove (see Appendix A.3.1) that the vague prior
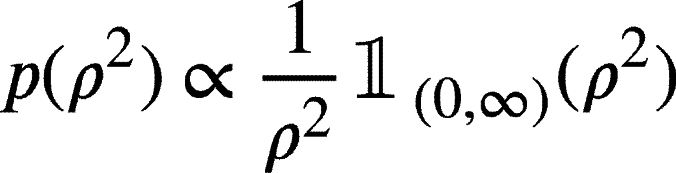
 yields the conditional posterior^4^

$$\rho^{2}|\sigma^{2},\nu ∼\text{Gamma}\left(\frac{J\nu }{2},\frac{J\nu }{2{\overset{ˆ}{\rho }}^{2}}\right),$$

where

$${\overset{ˆ}{\rho }}^{2}=\frac{J}{\sum_{j=1}^{J}\frac{1}{\sigma_{j}^{2}}}.$$

Unfortunately, the conditional posterior of *ν* is far more complicated, let be ω=ν/2, then

$$p(\nu |\sigma^{2},\rho^{2})\propto p(\nu )\frac{\omega^{J\omega }}{\Gamma^{J}(\omega )}{\left(\rho^{2}\right)}^{J\omega }\exp\left\{-J\omega \frac{\rho^{2}}{{\overset{ˆ}{\rho }}^{2}}\right\}\prod_{j=1}^{J}{\left(\sigma_{j}^{2}\right)}^{-\omega }.$$

This is an intricate expression, which gives little guide for the selection of a prior distribution for *ν* yielding a proper known distribution.

In general, it remains challenging to propose noninformative priors for the degrees of freedom of a distribution. In [51] the authors present different proposals for the degrees of freedom of a *t*-distribution under certain conditions. Notably, in [38], the authors present a process to build objective priors, which they called *Penalised Complexity* or PC priors. Let as it be, these approaches do not necessarily applied for the degrees of freedom of an inverse $\chi^{2}$ distribution. Thus, in this work we consider three different approaches to propose a prior distribution for *ν*.

#### 3.3.1 Estimating $\rho^{2}$ and *ν*: the hierarchical model with fixed $\overset{ˆ}{\nu }$

Because $\sigma_{j}^{2}|\nu ,\rho^{2}∼\text{Inverse-}\chi^{2}(\nu ,\rho^{2})$, we can use the method of moments to estimate $\rho^{2}$ and *ν*. Let be $E_{s^{2}}$ the average of the observed sample within-group variances, $s_{1}^{2},\ldots ,s_{J}^{2}$, and $V_{s^{2}}$ their variance, using the method of moments (see Appendix A.3.2), we get the following estimates

(2) $$\overset{ˆ}{\nu }=\frac{2{\left(E_{s^{2}}\right)}^{2}}{V_{s^{2}}}+4$$

and

(3) $${\overset{ˆ}{\rho }}^{2}=\left(\frac{2{\left(E_{s^{2}}\right)}^{2}+2V_{s^{2}}}{2{\left(E_{s^{2}}\right)}^{2}+4V_{s^{2}}}\right)E_{s^{2}}$$

Thus, the first option considered in this work is to fix the value of *ν* to its empirical estimator.

#### 3.3.2 Using a vague improper prior for *ν*

Setting *ν* to a fix value like $\overset{ˆ}{\nu }$ ensures us that the posterior distribution would exist for all the parameters, except *ν* which is no longer modeled as a random variable. This means that, setting the value of *ν* to a fix value, eliminates the uncertainty that we have on that parameter, and makes our model overconfident because it acts as if $\overset{ˆ}{\nu }$ would be the real value of *ν*. For this reason, the second approach in this work is to consider a vague prior for *ν*.

For modeling the degrees of freedom of a multivariate *t*-distribution, [52] proposed the prior $p(\nu )\propto {\left(\nu +1\right)}^{-3/2}$, while [53] proposed to use $p(\nu )\propto \nu^{-2}$. Thus, we proposed a prior for *ν* of the form , fixing the value of h>0. We have seen in simulations that large values of *h* tend to make each within-variance, $\sigma_{j}^{2}$, to concentrate in the observed sample variance $s_{j}^{2}$ at the cost of increasing the uncertainty in their estimates. Meanwhile, smaller values for *h* have the opposite effect, generating models that are closer to the case where a single common within-variance, $\sigma^{2}$, is considered for all the groups. However, even while it seems as a reliable approach in the simulations, we do not have any guarantee that the posterior distribution would exist using these improper priors. For example, the limit case h→0, corresponding with the prior , generates an improper monotonically increasing posterior for *ν*, which makes all the within-variances to concentrate in a common-variance quantity. In this work we present results for h=3,2,1.

Note that performing Gibbs sampler for this approach is still possible. To sample from the distribution of *ν*, we could use a grid of values and sample them with a probability proportional to the (conditional) posterior of those values. However, this requires the extra-effort of finding an appropriate grid.

#### 3.3.3 Using a regularizing prior for *ν*

Because setting the value of *ν* to a fix value, $\overset{ˆ}{\nu }$, eliminates the uncertainty on *ν*, and using an improper distribution does not give guarantee for the existence of the posterior distribution, the third option that we propose is to use a regularizing prior for *ν*.

A regularizing prior is a prior distribution whose parameters are learned from the data, which might prevent overfitting [39], [41]. With this approach, we maintain the uncertainty on *ν* with the guarantee of the existence of the posterior distribution. In this work, we consider an exponential distribution whose rate parameter is set at $1/\overset{ˆ}{\nu }$, but other distributions might be considered as well.

As commented previously, using Gibbs sampler is still feasible, but requires an extra-effort of finding an appropriate grid of values to sample from. However, we can use other Monte Carlo techniques to simulate from the joint posterior distribution. For this purpose, we used No U-Turn Sampler (NUTS) [54] which is a Hamiltonian Monte Carlo technique [55], implemented in the library PyMC (formerly PyMC3) [56].

### 3.4 Comparison of models

We implemented the discussed models for the variables selected in Section 2.3 and calculated the WAIC for each one of them. We present in Table 1 these values. We show in bold the lowest value of the WAIC for each variable, which corresponds with the preferred model according to this criterion.

**Table 1:** WAIC for each one of the models previously discussed and all the selected variable. We show in bold the model with the lowest WAIC value for each variable, being the preferred model according to this criterion.

**Table:** No Complete Hierarchical Hierarchical Pooling Pooling common *σ*^2^ fixed $\overset{ˆ}{\nu }$ Monthly Income 1382.01 1410.13 1386.44 1379.21 Percentage with Formal Debt -216.66 -183.14 -217.39 -221.02 Formal Debt 1795.12 1816.79 **1790.54** **1790.54** Percentage without Savings -189.07 -149.70 -191.04 -193.89 Yearly Savings 1515.78 1519.94 1518.12 **1512.98** Smoking -265.82 -217.48 -262.48 -267.08 Alcohol Consumption -244.94 -223.07 **-252.58** -250.27
**Table:** Hierarchical Hierarchical Hierarchical Hierarchical Exponential($1/\overset{ˆ}{\nu }$) *h* = 3 *h* = 2 *h* = 1 Monthly Income **1378.58** 1380.30 1380.15 1380.31 Percentage with Formal Debt **-221.04** -219.30 -220.08 -219.97 Formal Debt **1790.54** 1792.28 1791.51 1790.73 Percentage without Savings **-193.98** -191.80 -191.86 -192.60 Yearly Savings 1513.16 1513.94 1513.56 1514.96 Smoking **-267.70** -266.74 -266.99 -265.20 Alcohol Consumption -250.43 -247.72 -249.07 -252.85

More important that its use for model selection, the WAIC can help us for comparison of models. The objective is not to determine which is the correct model, a statement that is probably false for all the models, especially in the field of social science, but to determine which models can be *potentially feasible to explain the data*.

To answer this problem, a punctual value of the WAIC is not enough. Then, in Fig. 6, we present the credible intervals for the WAIC (at a 0.95 posterior probability) of the implemented models for the monthly income variable. The model with the lowest WAIC is when we model *ν* following an exponential distribution with rate parameter $1/\overset{ˆ}{\nu }$. However, all the hierarchical models that introduce a multilevel structure in the regional means and the within-region variances are feasible for explaining our data. Meanwhile, the WAIC of the models that assume a common within-variance $\sigma^{2}$ or complete pooling are far from the preferred one, so we cannot consider them as reliable models for our data.

**Figure 6:**
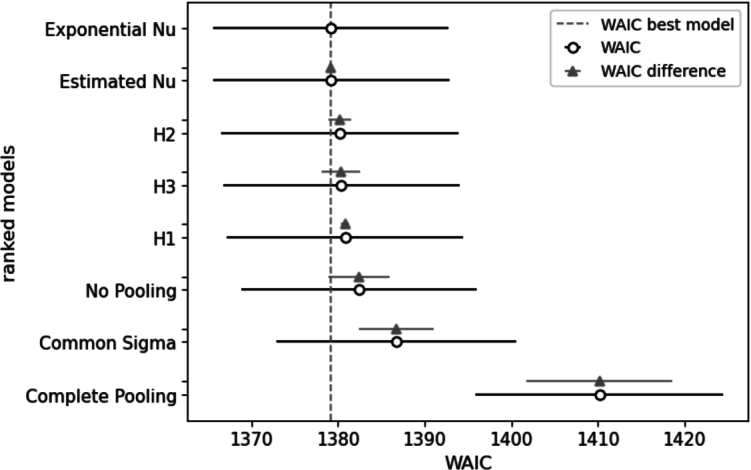
WAIC for the different models implemented for the monthly income variable. The WAIC can help us to determine plausible models for the studied phenomenon.

As it is usual now, Fig. 7 shows the results of the model with the lowest WAIC for the monthly income variable. On the left of the top row we present the regional mean of the monthly income, while the deviation within each region is presented on its right. We observe that the model can explain not only the observed average in each region, but also its variability. On the left of the bottom row we show the average monthly income per province in each one of the regions. An important difference from the complete pooling or no pooling models, is that the hierarchical model explicitly adds parameters that model the national behavior. For example, the parameter *μ* models the national average monthly income, whose posterior distribution is presented on the right of the bottom row in Fig. 7.

**Figure 7:**
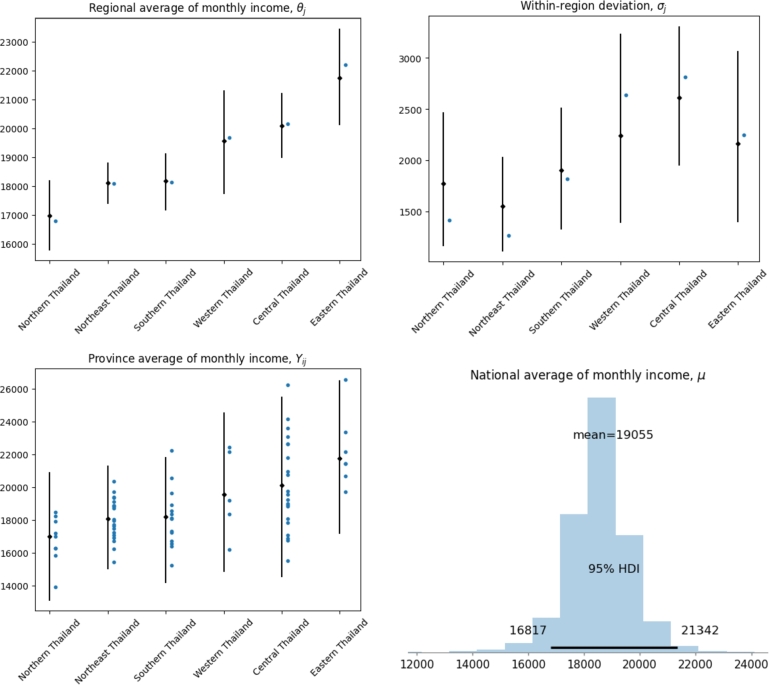
Results considering a hierarchical model. We allow, both the mean of each region and the within-region variance $\sigma_{j}^{2}$ to vary. We considered the prior $\sigma_{j}^{2}|\nu ,\rho^{2}∼\text{Inverse-}\chi^{2}(\nu ,\rho^{2})$. For *ρ*^2^ we used the vague prior , while $\nu ∼\text{Exponential}(1/\overset{ˆ}{\nu })$. On the top row we show the regional mean, *θ*_*j*_ (left) and the regional standard deviation, *σ*_*j*_ (right). On the bottom row we show the province mean (left) and the national average monthly income, *μ* (right).

Finally, to get some insight into the meaning of these estimations, Fig. 8 presents two maps of Thailand,^5^ the observed average income per province is presented on the left, while the regional average income is presented on the right.

**Figure 8:**
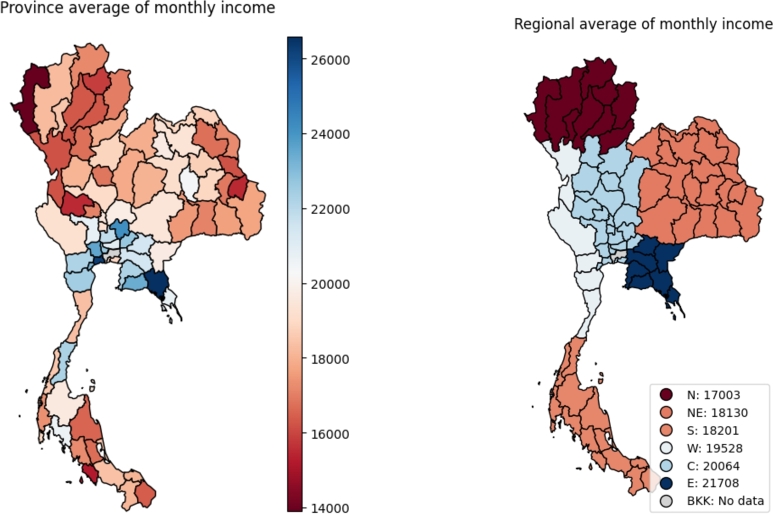
Left: Average monthly income per province. Right: Average regional monthly income.

## 4 Hierarchical model with two non-nested clusters: income per region and education level

In this section we extend our hierarchical model to estimate the income not only by region, but also by education level. From a statistical perspective adding the education level to our model means that we add another cluster to the model which do not hold a hierarchical structure with the first one. For this purpose, we have assigned the observations to one of three mutually exclusive groups according to the highest education of the people in the house. We have called this variable *education level*, whose possible values are low, mid or high, and whose assignation is done accordingly to the rule presented in Table 2.

**Table 2:** Education, education level and years of education.

Education	Years of education	Education level
Uneducated	0	Low
Kindergarten	0	
Pre-elementary school	3	
Elementary school	6	

Junior high school	9	Mid
Senior high school	12	
Vocational degree	14	

Bachelor degree	16	High
Post-graduate	19	

In Table 3, we present the percentage of the population belonging to the different levels of education, we can observe that approximately half of the population belongs to the mid education level, i.e. they completed elementary school but do not hold a bachelor or post-graduate degree, with the other two groups representing a significant percentage of the population each one. The percentage of the population with a low education level rounds 27% while the percentage for those with a high education level is around 22%. Thus, the amount of observations belonging to each group is large enough to achieve reliable results per education level.

**Table 3:** Proportion of observations according to the highest education in the house and its corresponding education level.

UN-EDU	KDG	P-ELEM	ELEM	JHS	SHS	VD	BD	PG
0.82%	0.03%	2.25%	23.65%	17.35%	23.94%	9.88%	20.97%	1.11%
			26.75%			51.17%		22.08%

Let be $Y_{ijk}$ the average income in the province *i*, belonging to region *j*, when the education level is equal to *k*. We maintain the hierarchical structure for both the regional mean and the within-region variance. But in this case, we allow the variance, $\sigma_{jk}^{2}$, to vary not only between regions but also between education levels. We now present our hierarchical model, whose Bayesian network is shown in Fig. 9:
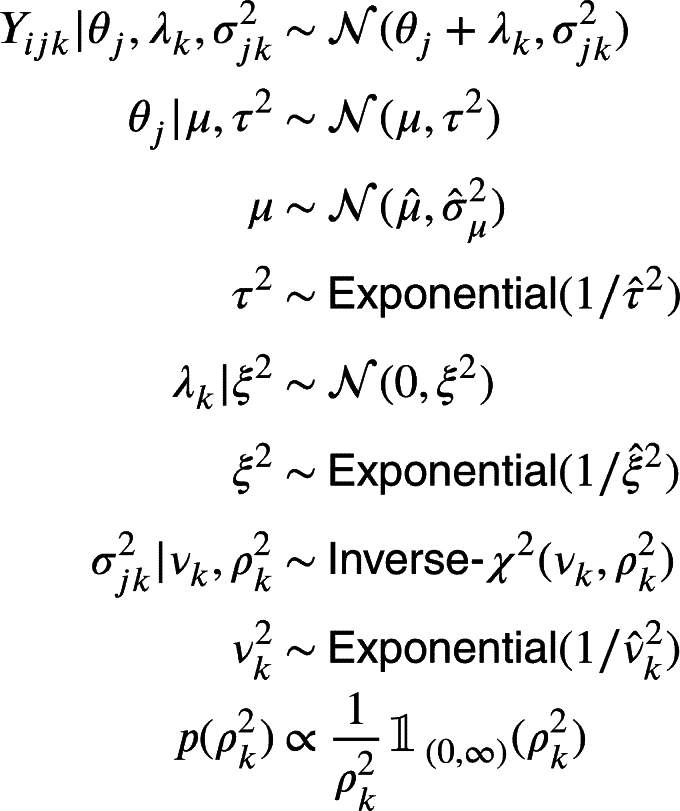
 We proceed to explain the different parts of this model and how the hyperprior distributions where setting.

**Figure 9:**
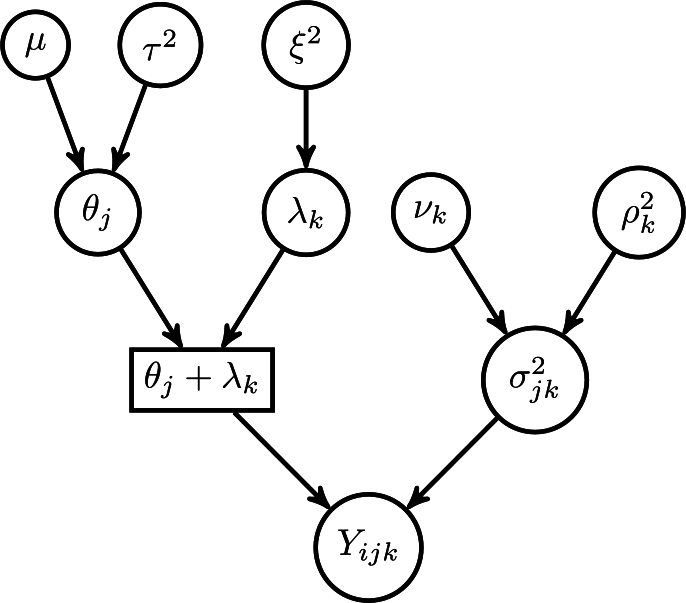
Hierarchical model with non-common *σ*^2^ and two non-nested clusters. Random variables are represented inside circles, while a square represents a deterministic relation.

*Likelihood.*  The first line of our model corresponds with the likelihood. We model $Y_{ijk}$ as a normal variable with variance $\sigma_{jk}^{2}$, and mean $\theta_{j}+\lambda_{k}$. The average monthly income of region *j* is still modeled by $\theta_{j}$, while $\lambda_{k}$ is interpreted as the additional income due to the education level.

*Prior distribution for*$\theta_{j}$*.*  For the regional average monthly income we use a normal distribution as before, with mean *μ* representing the average national monthly income, and variance $\tau^{2}$ representing the variance of the monthly income between regions.

*Prior distribution for μ.*  To establish the prior distribution for *μ* we use the hierarchical model proposed in Section 3.3. Then, we use the posterior sample of *μ*, being $\overset{ˆ}{\mu }$ its average and ${\overset{ˆ}{\sigma }}_{\mu }^{2}$ its variance.

*Prior distribution for*$\tau^{2}$*.*  Similarly to the prior distribution of *μ*. To establish the prior distribution for $\tau^{2}$ we use one more time the hierarchical model proposed in Section 3.3. Then, we use the posterior sample of *τ*, being $\overset{ˆ}{\tau }$ its average. Note that, in this way, we use previous simpler models as building blocks to construct the priors of more complex models.

*Prior distribution for*$\lambda_{k}$*.*  Consider the mean of $Y_{ijk}$, $\theta_{j}+\lambda_{k}$, and note that because $\theta_{j}|\mu ,\tau^{2}$ and $\lambda_{k}|\xi^{2}$ follow normal distributions, it can be written as

$$\theta_{j}+\lambda_{k}=\mu +\tau Z_{1}+\xi Z_{2},$$

where $Z_{1}$ and $Z_{2}$ are independent standard normal variables. From this expression, it is easy to observe that we have set the mean of $\lambda_{k}$ to zero to have an identifiable model. If, on the other hand, we introduce a non-zero mean for $\lambda_{k}$, we would not have any way to distinguish between both *μ* and this new hyperparameter.

For a fix *k*, we can estimate $\lambda_{k}$ as follows. We first implement the hierarchical model proposed in Section 3.3 but only for those observations whose education level equals *k*, let be $\mu_{k}$ the national monthly income for this model. On the other hand, we implement the same hierarchical model for all the observations (note that this is the model used for the prior specification of both *μ* and $\tau^{2}$). Thus, a punctual estimator for $\lambda_{k}$, denoted as ${\overset{ˆ}{\lambda }}_{k}$, is given by the posterior mean of the variable $\lambda_{k}=\mu_{k}-\mu$.

*Prior distribution for*$\xi^{2}$*.*  Because $\xi^{2}$ represents the variance of $\lambda_{1},\ldots ,\lambda_{K}$, we can estimate it with the variance of ${\overset{ˆ}{\lambda }}_{1},\ldots {\overset{ˆ}{\lambda }}_{K}$, denoted as ${\overset{ˆ}{\xi }}^{2}$. Then, for the prior distribution of $\xi^{2}$ we use an exponential distribution with rate $1/{\overset{ˆ}{\xi }}^{2}$.

*Prior distribution for*$\sigma_{jk}^{2}$*.*  For this model, we assume that the within-region variance can vary not only between regions but also between education levels. For the prior of $\sigma_{jk}^{2}$ we use the usual inverse-$\chi^{2}$ distribution presented in Section 3.3.

*Prior distribution for*$\nu_{k}$*.*  For a fix *k*, we estimate $\nu_{k}$ through Equation (2) considering those observations whose education level equals *k*. An exponential distribution with rate parameter equal to $1/{\overset{ˆ}{\nu }}_{k}$ is used as proposed in Section 3.3.3.

*Prior distribution for*$\rho_{k}^{2}$*.*  For $\rho_{k}^{2}$, we use the vague prior , which yields a proper posterior distribution.

We show in Table 4 the WAIC with and without considering the education level. We observe that considering the education level leads to a huge reduction of the WAIC, preferring the model that incorporates both clusters. Note that the values of the WAIC are around three times those presented in Table 1 for the monthly income variable. However, these quantities are not comparable. The reason is that when the education level is considered we have the union of three datasets, one for each education level for the 76 provinces. Then, the model which incorporates the education level has three times the number of observations of the models that do not incorporate it, making the WAIC incomparable between both set of models.

**Table 4:** WAIC without considering the education level, and when both region and education level are added to the model.

	Without	With region and
	education level	education level
Monthly Income	4662.59	**4096.79**

In Fig. 10 we present the estimated monthly income at a national (left) and regional level (right). A close inspection reveals a similar distribution for the national average compared with the results presented in Fig. 7. However, the regional averages show more overlap, this indicates that, once we consider the education level, the region has less impact in the income. This might be the result of a mediation relation, in which education level acts as a mediator between the region and the income. This same phenomenon can be observed in Fig. 11 with more color-homogeneous maps for each education level, but with large differences between them. For those readers interested in causal inference, we recommend [50], [57], [58].

**Figure 10:**
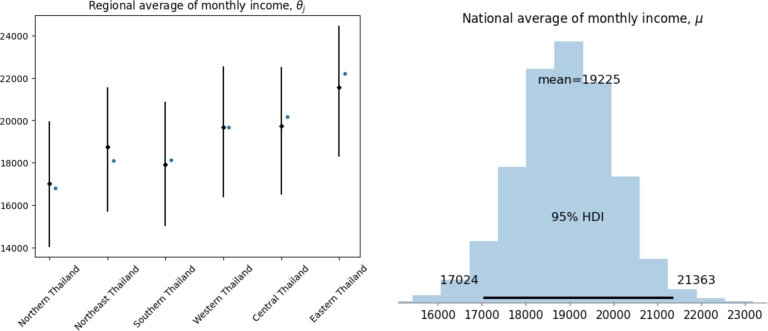
Estimated monthly income, using a hierarchical model that incorporates the region and the education level. Left: Regional average monthly income. Right: National average.

**Figure 11:**
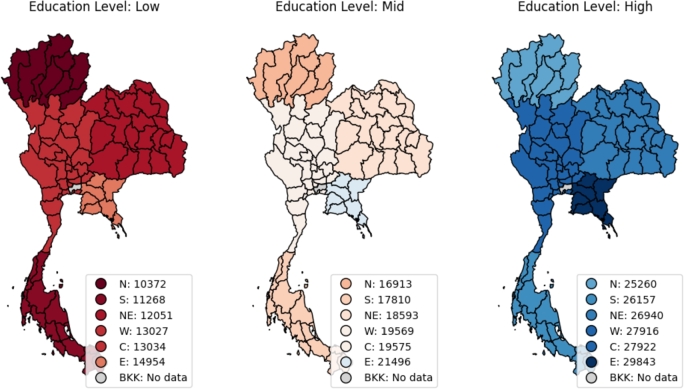
Average monthly income per region and education level.

In Appendix B we present supplementary Figures for this model.

## 5 Bayesian hierarchical regression: income considering years of formal education

### 5.1 National model and separate models

Instead of considering the education as a categorical variable, we can approximate the years of formal education received, using the rule presented in Table 2, denote this new variable by *X*. Then, we can implement a regression model that estimates the income taking as input the years of education.

According to our proposed procedure, before presenting the full hierarchical model, we first consider two simple models. The complete pooling model and separate independent models for each region. These models are represented graphically in Fig. 12.

**Figure 12:**
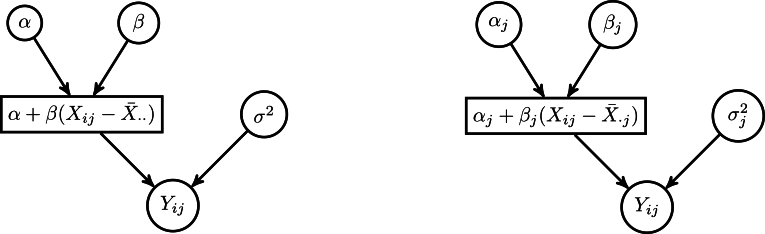
Graphical representation of regression models. Left: National model, where only one regression function is considered for the all the regions. Right: No pooling model, where a regression model is implemented for each region independently of the others.

#### 5.1.1 National model

Let be $X_{ij}$ the average years of formal education in the province *i* belonging to region *j*. The complete pooling model means the implementation of a single regression function that models the national relation between the income and the years of formal education. With this simple model, we can use vague priors for the parameters without harmful. We introduce now our national model:
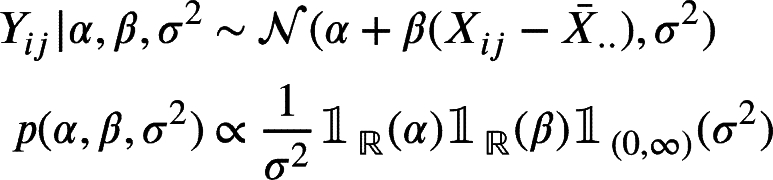


The expected income $Y_{ij}$ is modeled through the regression function that takes as input the years of education $X_{ij}$, for simplicity we have considered a linear function for the regression, given by $\alpha +\beta (X_{ij}-{\overset{¯}{X}}_{\cdot \cdot })$, where ${\overset{¯}{X}}_{\cdot \cdot }$ is the average years of formal education between all the provinces, that is

$${\overset{¯}{X}}_{\cdot \cdot }=\frac{\sum_{j=1}^{J}\sum_{i=1}^{n_{j}}X_{ij}}{n},$$

where $n=\sum_{j=1}^{J}n_{j}$. Note that *α* represents the average national income when the years of education equal the national average, while *β* represents the amount of income added per year-of-education.

We present in Fig. 13 the posterior distributions for *α* and *β*, Fig. 14 presents the estimated regression function, we also present credible bands for the regression function and the income, both bands are calculated at a 0.95 posterior probability.

**Figure 13:**
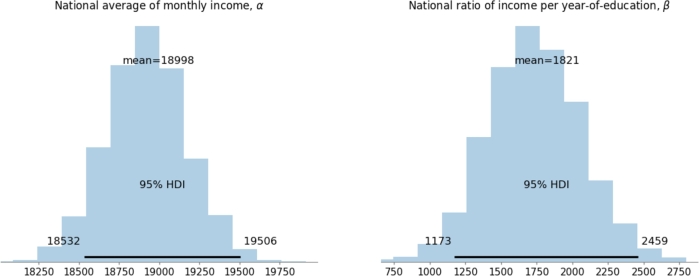
Left: National average monthly income when the years of formal education equal the national average. Right: National ratio income per year-of-education.

**Figure 14:**
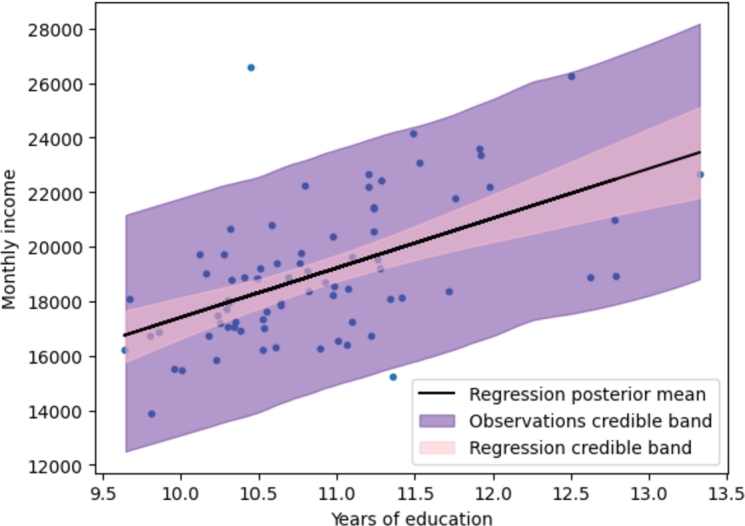
National regression model for the monthly income given the years of formal education.

#### 5.1.2 Separate models

Instead of considering just one regression function for all the regions, we can estimate a regression function for each one of the regions, independent from the others. Similarly to the national regression model, for each one of these models we can use vague priors for the parameters without harmful:
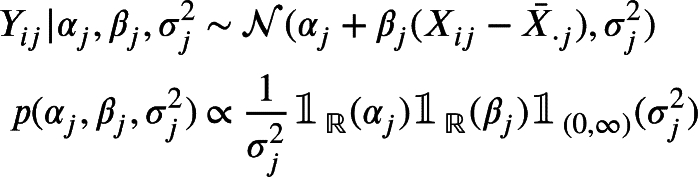


We consider a linear function for the regression in each region *j*, given by $\alpha_{j}+\beta_{j}(X_{ij}-{\overset{¯}{X}}_{\cdot j})$, where

$${\overset{¯}{X}}_{\cdot j}=\frac{\sum_{i=1}^{n_{j}}X_{ij}}{n_{j}}$$

is the average years of formal education in the region *j*. Note that $\alpha_{j}$ represents the average income in the region when the years of education equal the regional average, while $\beta_{j}$ represents the amount of income added per year-of-education in the region.

On the left of Fig. 15, we present the average monthly income for each region when the years of education are equal to the regional mean, $\alpha_{j}$, with their respective credible intervals of 0.95 posterior probability and the observed average income in each region. Analogously, on the right we present the ratio of income per year-of-education, $\beta_{j}$. Because this model assumes that the regions do not share any information, we observe large credible intervals, for some regions like Southern Thailand or Eastern Thailand these intervals even include negative values, which seems implausible. Moreover, as we pointed before, pretending that each region is independent for the others seems unrealistic. Therefore, we introduce the hierarchical regression model as a compromise between a single regression model and an independent regression model for each region.

**Figure 15:**
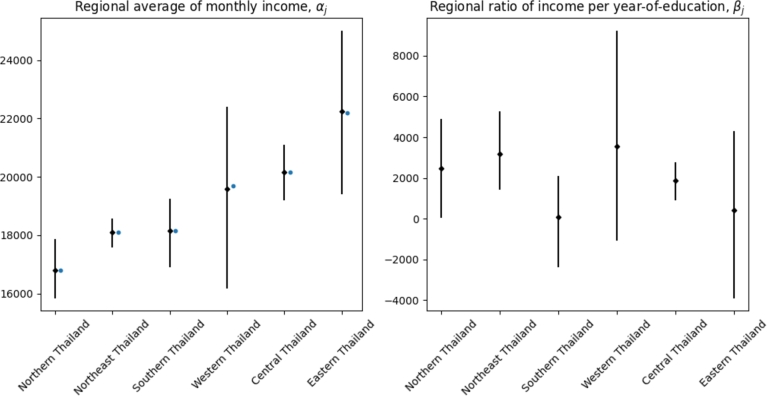
Left: Regional average monthly income when the years of formal study equal the regional average. Right: Regional ratio income per year-of-education.

### 5.2 Bayesian hierarchical regression varying intercepts

We already observed in Section 3.3 that adding a common structure to the region average income and to the within-regions variance results in a better model, so we can implement a model with those characteristics for the regression task. This model is represented graphically in Fig. 16.

**Figure 16:**
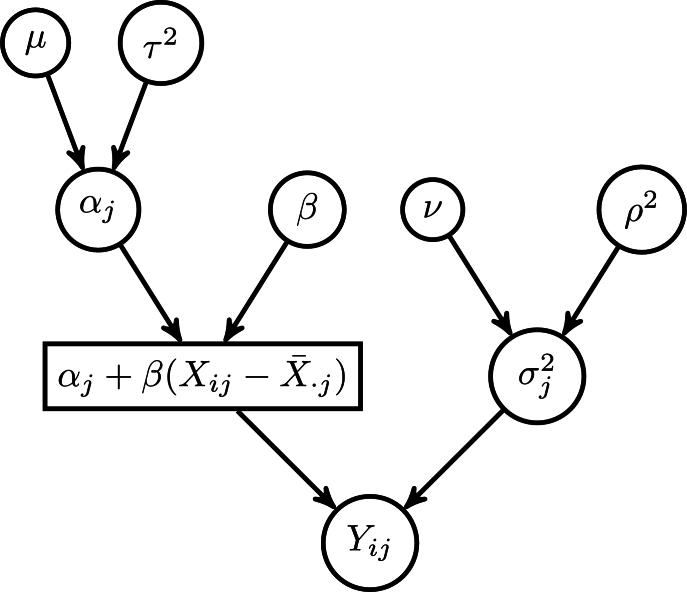
Graphical representation of the Bayesian hierarchical regression model, varying intercepts but considering a common slope for all the regions.

We now present our hierarchical model for the regression task:
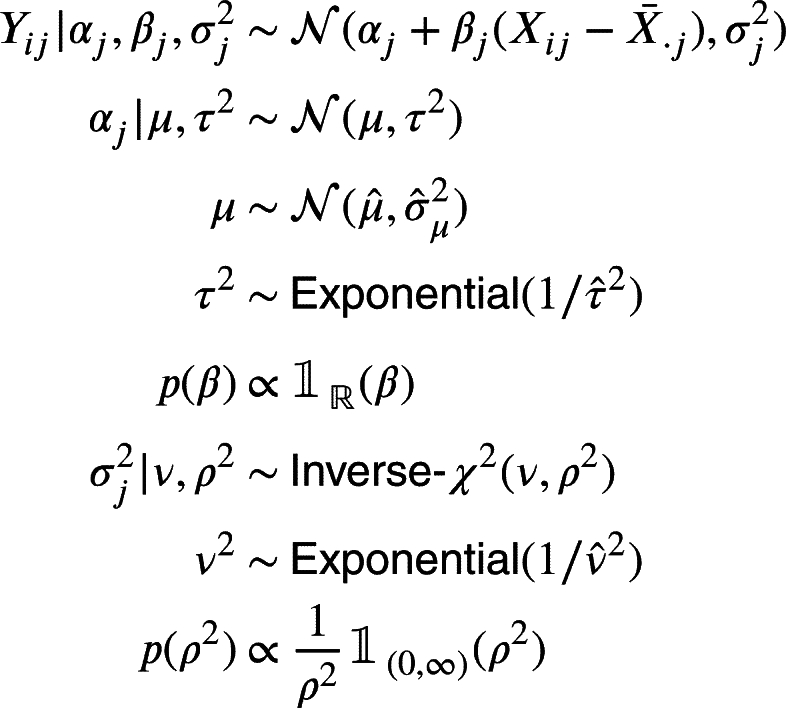


Many parts of this model have been inherited from our previous hierarchical models. Then, we explain only the prior distributions of the hyperparameters that changed from the previous models.

*Prior distribution for μ.*  Note that *μ* has a similar interpretation than the intercept parameter in the national regression model. Thus, we set $\overset{ˆ}{\mu }$ and ${\overset{ˆ}{\sigma }}_{\mu }$ to the mean and deviation (respectively) of the intercept parameter in the national regression model.

*Prior distribution for*$\tau^{2}$*.*  Note that $\tau^{2}$ models the variance between $\alpha_{1},\ldots ,\alpha_{J}$. Then, we can estimate this quantity from the no pooling regression model. To do so, we first compute the average for the intercept parameter for each region, and then take the variance between these values.

In Fig. 17 we present analogous graphs of those presented in Figure 13, Figure 15. Note that the distribution of *β* includes negative values, which could indicate that different slopes should be preferred instead of a common national parameter.

**Figure 17:**
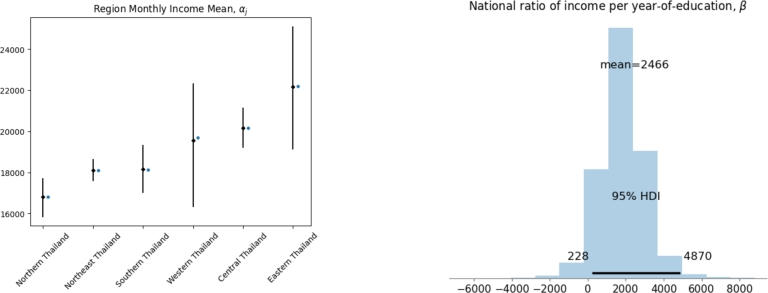
Left: Regional average monthly income when the years of formal study equal the regional average. Right: National ratio income per year-of-education.

### 5.3 Bayesian hierarchical regression varying intercepts and slopes

Because a common slope for all the regions seems inappropriate for this case, we now present a model that implements a hierarchical model on both parameters, the intercept and the slope. This model is represented graphically in Fig. 18.

**Figure 18:**
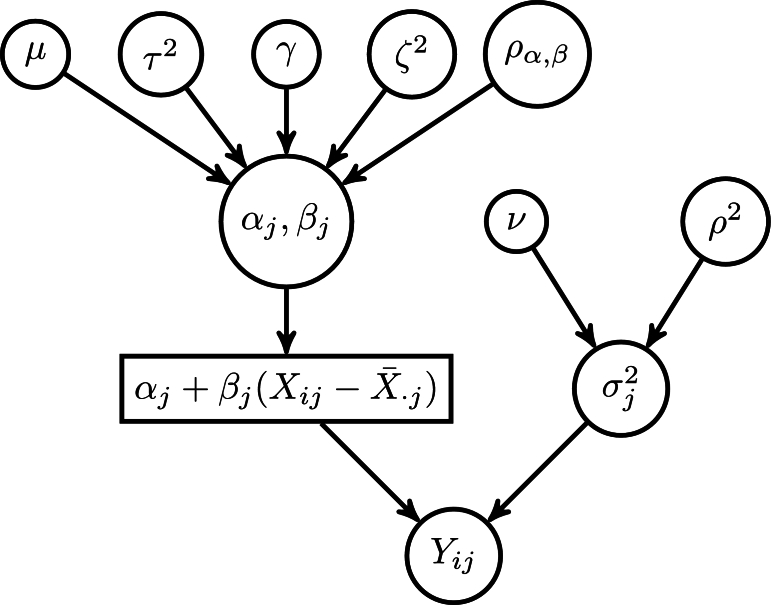
Graphical representation of the hierarchical Bayesian regression model, varying intercepts and slopes.

Instead of just incorporating a normal distribution for the slopes into the previous model, we model the intercepts and slopes through a multivariate normal distribution, allowing them to covary. Then, $\alpha_{j}$ and $\beta_{j}$ will follow a multivariate normal distribution with mean (μ,γ) and a matrix of variances and covariances

$$S=\left(\begin{array}{cc} \tau^{2} & \tau \zeta \rho_{\alpha ,\beta }\\ \tau \zeta \rho_{\alpha ,\beta } & \zeta^{2} \end{array}\right),$$

which can be written as

$$S=\left(\begin{array}{cc} \tau & 0\\ 0 & \zeta \end{array}\right)R\left(\begin{array}{cc} \tau & 0\\ 0 & \zeta \end{array}\right),$$

where

$$R=\left(\begin{array}{cc} 1 & \rho_{\alpha ,\beta }\\ \rho_{\alpha ,\beta } & 1 \end{array}\right)$$

is the correlation matrix.

We are now ready to present our hierarchical model:
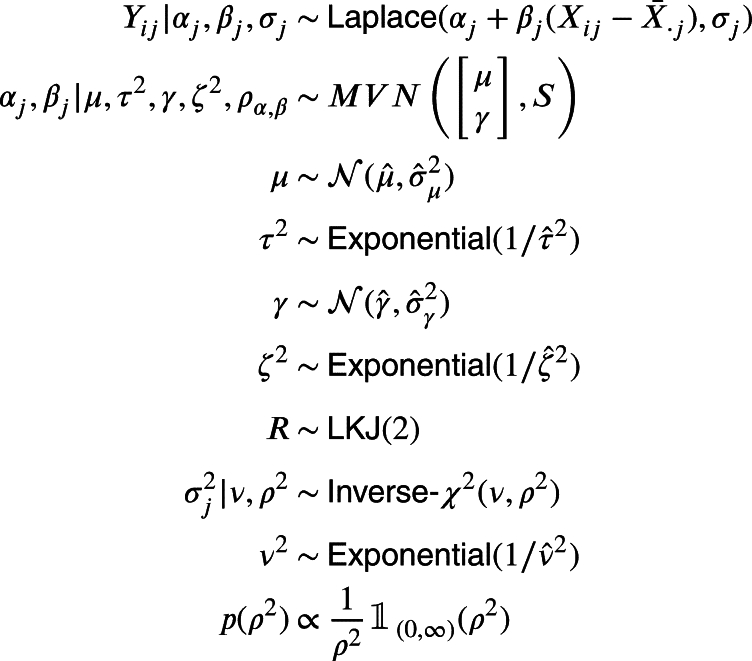


*Likelihood.*  In this model we do not consider anymore a normal likelihood for our data. Instead, we consider a Laplace distribution. The density of a random variable *Y* that follows a Laplace distribution with parameters *μ* and *σ* is given by

$$p(Y|\mu ,\sigma )=\frac{1}{2\sigma }\exp\left(-\frac{\left|Y-\mu \right|}{\sigma }\right).$$

This change in the likelihood is analogous to median regression in which the absolute errors are minimized, and thus corresponding to a robust regression model. As mentioned in [59], this can be generalized to other quantiles using the asymmetric Laplace distribution [60], [61].

We can observe in Fig. 19 and Table 5 that with this change none of the credible intervals (calculated at a 0.95 posterior probability) for the ratio of income per year-of-education includes negative values, even while a constraint of positive values for these parameters was not incorporated in the model. If instead of a Laplace distribution, we would have considered a normal distribution for the data, some of these intervals would include negative values, which seems unfeasible to explain.

**Figure 19:**
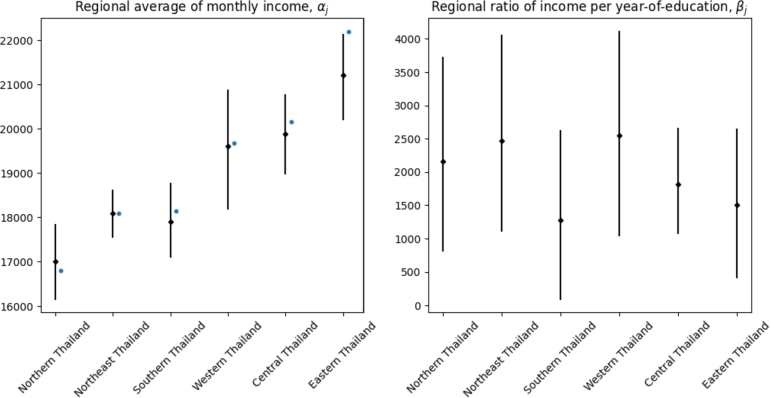
Left: Regional average monthly income when the years of formal study equal the regional average. Right: Regional ratio income per year-of-education.

**Table 5:** Amount of monthly income added per each year-of-education. We present the posterior mean for the national level and for each one of the regions, as well as a credible interval of 0.95 posterior probability.

	Amount of monthly income
	added per year-of-education (THB)

National level	1880; (1322, 2443)

Northern Thailand	2159; (814, 3719)
Southern Thailand	2466; (1111, 4056)
Western Thailand	1278; (94, 2613)
Eastern Thailand	2545; (1051, 4109)
Northeast Thailand	1818; (1076, 2648)
Central Thailand	1509; (413, 2642)

*Prior distribution for γ.*  Analogously to the prior distribution for *μ*, we set $\overset{ˆ}{\gamma }$ and ${\overset{ˆ}{\sigma }}_{\gamma }^{2}$ to the mean and variance of the posterior distribution for the slope parameter in the national regression model.

*Prior distribution for*$\zeta^{2}$*.*  To set the prior of $\zeta^{2}$ we follow the same strategy used for the prior of $\tau^{2}$. That is, we calculate the posterior mean of the slopes for the separate independent regression models, and then we set ${\overset{ˆ}{\zeta }}^{2}$ to the variance of these posterior means.

*Prior distribution for R.*  For the prior of *R* we consider the LKJ distribution [62], which is a distribution over all positive definite correlation matrices where the shape is determined by a single parameter, η>0. Setting η=1 results in a uniform distribution over all the correlations $\rho_{\alpha ,\beta }$, however setting η=2 is an alternative that has been considered in the literature [39], [63], [64], [65] to define a weakly informative prior over $\rho_{\alpha ,\beta }$. It implies that $\rho_{\alpha ,\beta }$ is near zero, reflecting the prior belief that there is no correlation between intercepts and slopes.

In Fig. 19 we present analogous graphs of those presented in Fig. 13. Comparing the credible interval of the slopes, we can observe not only narrower intervals, but also that they contain only positive values, which means that every extra year of education generates a higher income. The credible intervals for the slopes and their posterior mean are also shown in Table 5. The fact that these intervals overlap suggests that this increment in the income is similar for all the regions.

With our hierarchical model, we can estimate the national average income when the years of education equal the national average years, which is modeled by *μ*, and whose posterior distribution is shown on the left of Fig. 20. On the right side we present the posterior distribution of *γ*, which models the national ratio of income-per-year of education.

**Figure 20:**
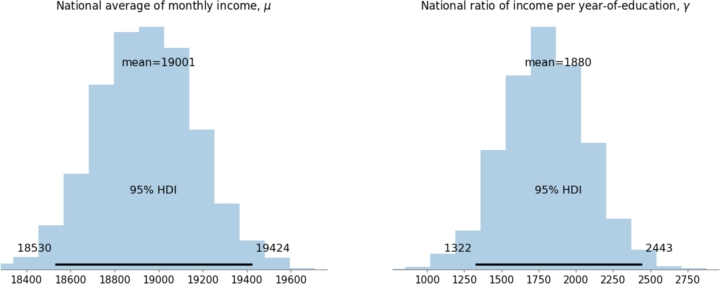
Left: National average monthly income. Right: National ratio of income per-year-of-education.

We show in Fig. 21 the estimated regression models with credible bands for the regression functions and the province monthly income, both bands are calculated at a 0.95 posterior probability. In Fig. 22 we show the joint posterior of the intercepts and slopes. On the left side we show the posterior mean of each pair $\left(\alpha_{j},\beta_{j}\right)$ and compare them to the posterior mean of the pair (α,β) for the national regression model. Similarly, on the right side we present our estimates for the slopes and intercepts, and compare them with the extreme case of considering separate independent models for each region. We can observe how the estimators are closer to more probable regions when we impose a hierarchical structure.

**Figure 21:**
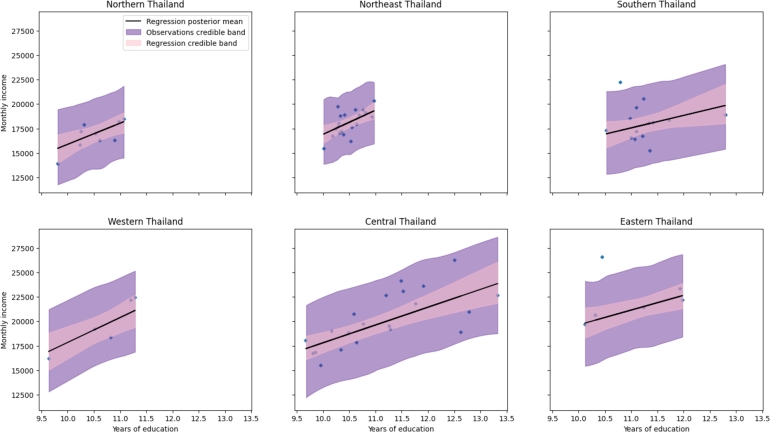
Regional regression models for the monthly income given the years of education, we impose a hierarchical model for the intercepts and the slopes, allowing them to covary.

**Figure 22:**
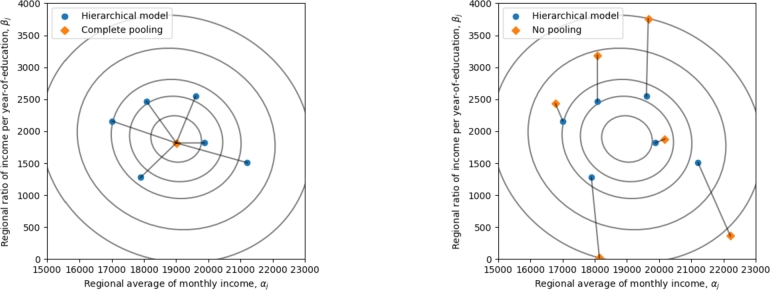
Joint posterior of the slopes and intercepts. Left: We show the posterior mean of each pair (*α*_*j*_,*β*_*j*_) and compare them with the posterior mean of the pair (*α*,*β*) for the national regression model. Right: We present our estimates for the intercepts and slopes and compare them with the estimators for separate independent models for each region.

Finally, we show in Table 6 and Fig. 23 the WAIC for all the regression models. For comparison purposes, we also present in Table 6 the WAIC for the preferred model without a covariate variable (see Table 1). Note that the model that allows variation between intercepts with a common slope is as feasible as the model that allows different intercepts and slopes. We observe that, except for the national regression model, all the regression models present a lower WAIC value that the model without covariables, making them more reliable models accordingly to this criterion.

**Table 6:** WAIC for the models of regression. In the first cell we present the lowest value in Table 1 for the models without a covariate.

	Without	National	Separate	Varying *α*_*j*_,	Varying *α*_*j*_,
	Covariable	Model	Models	Common *β*	Varying *β*_*j*_
Monthly Income	1378.58	1387.08	1358.77	1354.37	**1353.82**

**Figure 23:**
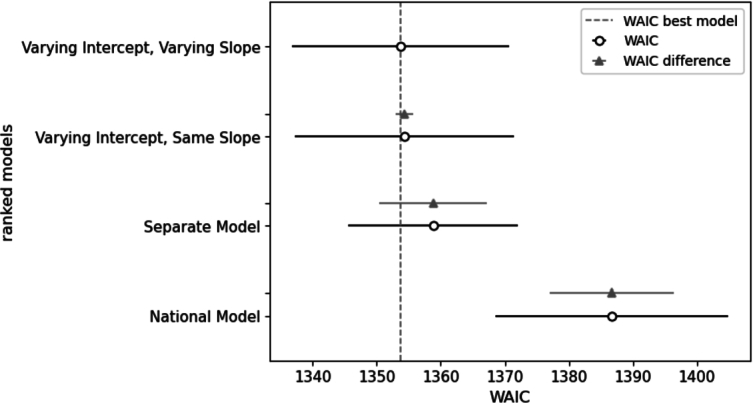
WAIC for the Bayesian hierarchical regression models.

## 6 Discussion of results

While this work is devoted to the analysis and implementation of Bayesian hierarchical models, we first introduced the extreme cases of no pooling and complete pooling models, corresponding to one-shirt-size and custom-made policies. We observed that none of these models was able to explain satisfactory our data, presenting serious drawbacks. For instance, in the case of separate models for each region, we observed large credible intervals, especially for regions with a few number of provinces. On the other hand, when we fit a single common model for all the regions, the uncertainty in the estimations was reduced dramatically since we were using all the observations to estimate them. However, we observed that in this case, the variance within the regions was overestimated. This awkward behavior is because, now that all the regions share the same mean, the only way to explain the variation in the observations is overestimating the variance. After analyzing these models, we introduced the hierarchical models as a better approach to explain our data.

For simple Bayesian hierarchical models, we took advantage of the conjugacy property of the proposed distributions to easily derive the full conditional posterior distributions of the parameters, which allows the implementation of simple MCMC techniques such as Gibbs sampling [47]. Furthermore, many of these distributions are well-known which makes even simpler the simulation from the posterior distribution of the whole set of parameters.

However, attempting to calculate the exact posterior distribution of the parameters for more complex hierarchical models might be extremely challenging. And, even if it is possible to obtain a close form for the full conditional posterior distributions, it might not follow a well-known distribution, which would represent a considerably drawback for the implementation of more complex and realistic hierarchical models since it would increase the computational resources needed and the time for their implementation. For example, as commented in Section 3.3, it is still possible to use Gibbs sampling when we consider an improper distribution for the degrees of freedom *ν*, with the extra-effort of finding an appropriate grid, which would be reflected in a lower computational performance of the model. Fortunately, now there is a wide list of Bayesian software programs [23] and alternative Monte Carlo techniques, such as Hamiltonian Monte Carlo [54], [55], that makes possible to implement more intricate Bayesian models.

In Section 4, we introduced the education level into our hierarchical model, creating a model with two non-nested clusters. It is important to notice that, since each new variable added to the model requires the specification of its prior distribution and its hierarchical structure, as well as to be sure that the parameters of the model remain identifiable, it remains extremely challenging to introduce several variables into the hierarchical models, which introduces an important drawback considering the multidimensional problem of poverty [3], [4].

While some distributions in our hierarchical models where chosen to get a conjugate model, a strong point of the Bayesian models is that their allow easily to incorporate other distributions that reflect better our knowledge of the phenomenon, allow a suitable interpretation, or to achieve robust inferences. As instance, we set the parameters related with the variance of a distribution to follow an exponential distribution for its interpretability, simplicity and the guarantee of the existence of their posterior distribution. Thus, if the data does not follow the assumptions reflected in the proposed distributions, they can be replaced for more appropriate ones. For example, at the end of Section 5 we abandoned the normality assumption of the data and imposed a Laplace distribution to achieve more robust results.

For the comparison of the models, alongside the analysis and discussion of the results, we used the Widely Applicable Information Criterion (WAIC) [43], [44]. We found that Bayesian hierarchical models performed better than both complete pooling (single policy) and no pooling models (custom-made policy), which is reflected in lower WAIC values.

It is worth to note that we did not consider the underlying spatial structure between the provinces, which represents a limitation for our models. For example, a province at the border of its region might be more similar to other provinces that could belong to other regions, than to the rest of the provinces within its own region.

A central concept to justify the use of hierarchical model is what is known in probability and statistics as *exchangeability*. As commented in [42], when there is no information to distinguish any of the parameters of the model from the others, and no ordering or grouping of the parameters can be made, one must assume that the parameters are exchangeable in their joint distribution. In practice, ignorance implies exchangeability. Generally, the less we know about a problem, the more confidently we can make claims of exchangeability. However, often observations are not fully exchangeable, but are partially or conditionally exchangeable. If observations can be grouped, we may make a hierarchical model, where each group has its own submodel. When assuming exchangeability we assume there are no important covariates that might form the basis of a more complex model. In such situation, we can expand the framework of the model to be exchangeable in the observed data and covariates, for example using a hierarchical regression model.

Therefore, we have implicitly considered that the provinces are exchangeable within each region given the variables considered so far, even while in reality there are differences between them that we did not consider. If such differences are significant, then the exchangeability assumption within the regions would be violated and a hierarchical model that did not consider such difference would be inappropriate.

## 7 Conclusions

One-shirt-size policy cannot handle poverty issues well since each area has its unique challenges, while having a custom-made policy for each area separately is unrealistic due to limitation of resources as well as having issues of ignoring dependencies of characteristics between different provinces. In this work we presented and discussed several Bayesian hierarchical models which can potentially explain the data regarding income and other poverty-related variables in the multi-resolution governing structural data of Thailand. These models present an optimal solution to two extremes – complete pooling and no pooling. That is, they are a good trade-off when independence between regions or groups is difficult or implausible to justify, but a common model for all the regions presents a poor performance. In such case, we introduced a common structure to the parameters of the model through informative priors and the use of hyperparameters, allowing variation in the estimation of the regions' parameters without making them completely independent.

Before starting directly with intricate hierarchical models, our proposed approach is to first introduce simple models which are later used as building blocks for the prior specification of the hyperparameters in more complex hierarchical models. In this way, hierarchical models allow the inference of prior distributions from data rather than requiring them to be specified using subjective information [40], which might be challenging for some parameters in high levels of the hierarchical model. Furthermore, in hierarchical models where priors depend on hyperparameter values that are data-driven avoids the direct problems of linked to double-dipping [23], without being overwhelmed with the problems of overfitting. After analyzing the results of simpler models and discussing their drawbacks to properly explain the data, we encouraged the introduction of hierarchical structures explaining how to design hierarchical models as long as it was suggested by the data.

By adding the year-of-education variable, the hierarchical models enrich their performance of variable explanation. We observed that having a higher education level increases significantly the households' income for all the regions in Thailand, reflecting the important impact of the education in the income, always showing a positive relation. Due to the multilevel structure of our models we were able to estimate the average income for each education level and the ratio of income per year-of-education, at regional and national levels. It is important to mention that the impact of the region in the households' income was almost vanished when education level or years of education were considered. Therefore, education might have a mediation role between regions and the income.

From the theoretical side, we presented a way to estimate the scale parameter and the degrees of freedom for an inverse $\chi^{2}$ distribution using the method of moments. We also proposed some vague priors for the degrees of freedom of the distribution for which, to the best of our knowledge, there is no literature presenting an alternative approach for this problem, remaining as an open problem to deduce a noninformative prior for the degrees of freedom with the guarantee of the existence of its posterior distribution.

We also encouraged the use of Bayesian networks for two main reasons, which were extensively exploded in this work. First, for their ability to visualize easily the hierarchical structures and relations between the variables, which helps in the interpretation and understanding o the models. Second, because they allow us to use *d*-separation rules [48] to deduce the conditional independence between the parameters, which could help when calculating the full conditional posterior distribution of the parameters.

Finally, we would like to emphasize that poverty is a serious issue in many developing countries, thus, statistical studies are badly needed to help the governments cope with poverty. Our work can serve as a guideline for other countries that require the Bayesian hierarchical approach to model their variables and get insight from data.

## Note

Codes to reproduce our results are available in https://github.com/IrvingGomez/BayesianHierarchicalIncome.

## CRediT authorship contribution statement

**Irving Gómez-Méndez:** Writing – review & editing, Writing – original draft, Software, Methodology, Investigation, Formal analysis, Data curation, Conceptualization. **Chainarong Amornbunchornvej:** Writing – review & editing, Writing – original draft, Supervision, Resources, Project administration, Conceptualization.

## Declaration of Competing Interest

The authors declare that they have no known competing financial interests or personal relationships that could have appeared to influence the work reported in this paper.

## Figures and Tables

**Figure 24:**
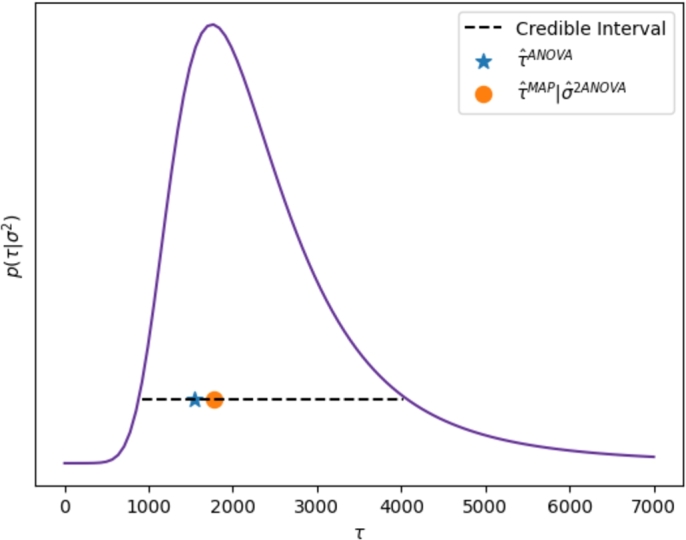
Posterior distribution of *τ* conditional on *σ*^2^. We have set *σ*^2^ to its empirical estimator. We show the empirical estimator for *τ* based on the ANOVA and the mode of the distribution. Both estimators are presented on an interval of approximately 0.95 posterior probability, we calculate this interval based on the asymptotic behavior of the posterior distribution.

**Figure 25:**
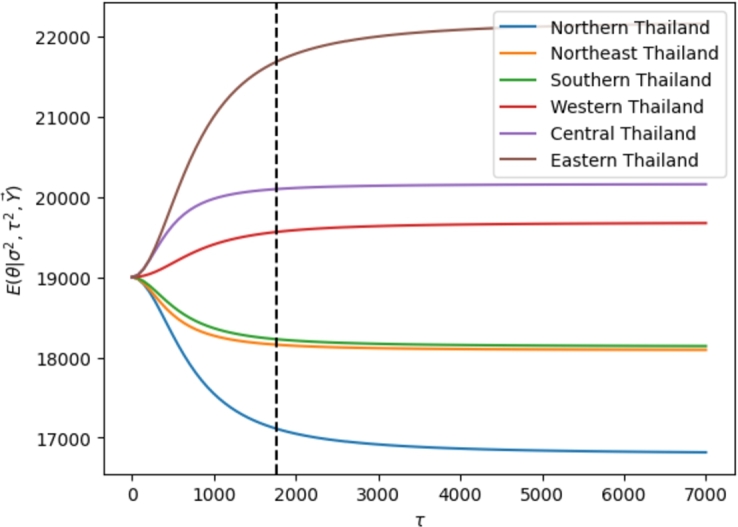
Conditional posterior mean for *θ*_*j*_, $E(\theta_{j}|\tau ,\sigma^{2},Y)$ (*j* = 1,…,*J*) as function of *τ*, setting *σ*^2^ to ${\overset{ˆ}{\sigma }}^{2\text{ANOVA}}$. The vertical dashed line is plot on ${\overset{ˆ}{\tau }}^{\text{MAP}}$.

**Figure 26:**
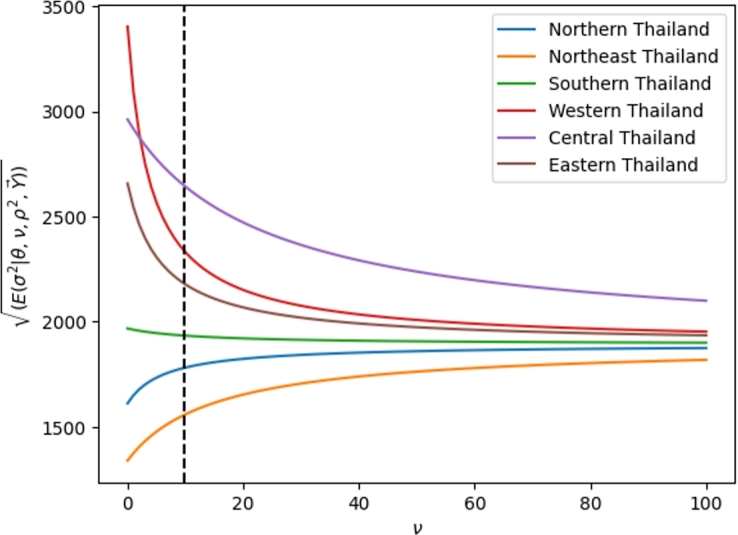
Square root of the conditional posterior mean for $\sigma_{j}^{2}$, $\sqrt{E(\sigma_{j}^{2}|\theta ,\nu ,\rho^{2},Y)}$ (*j* = 1,…,*J*) as function of *ν*, setting *ρ*^2^ to ${\overset{ˆ}{\rho }}^{2}$ and substituting *v*_*j*_ for the observed variance in the region. The vertical dashed line is plot on $\overset{ˆ}{\nu }$.

**Figure 27:**
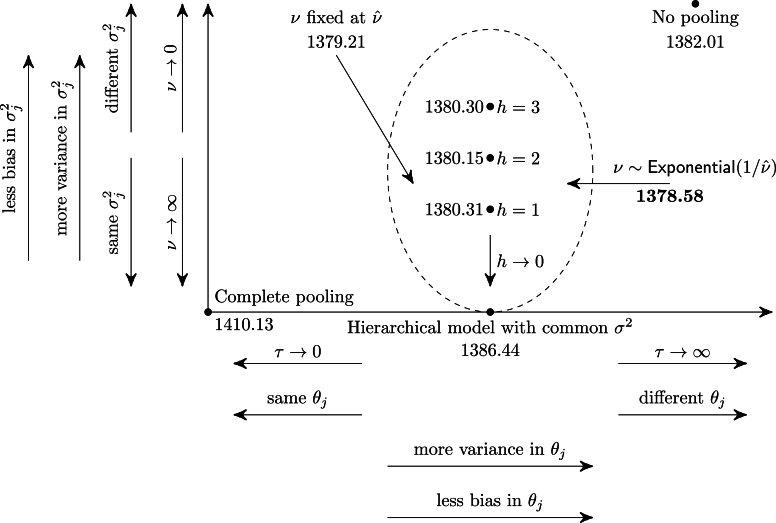
Relation between models and the influence of the different parameters in the qualitative characteristics of the models. Next to each model we present its corresponding WAIC value for the monthly income, presented in Table 1.

**Figure 28:**
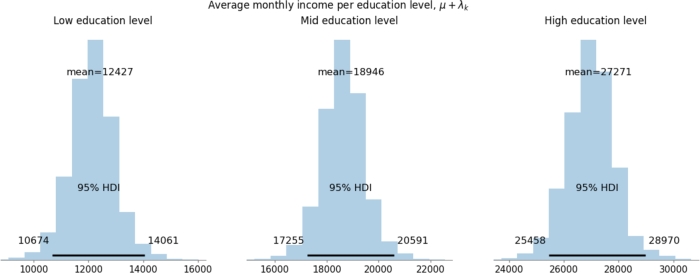
Average income per education level.

**Figure 29:**
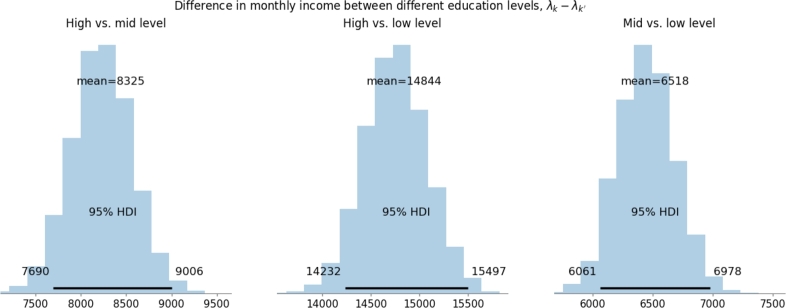
Difference between average income per education level.

**Figure 30:**
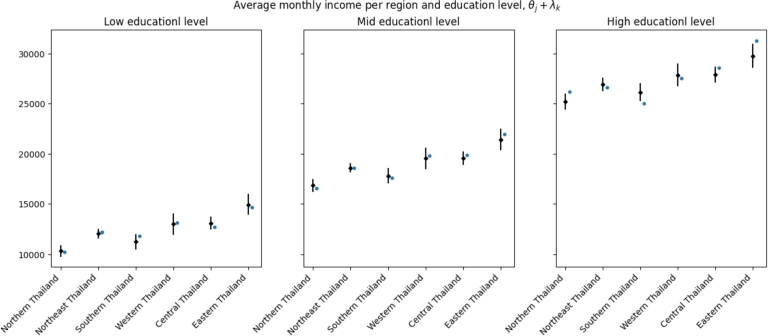
Average monthly income per region and education level.

**Figure 31:**
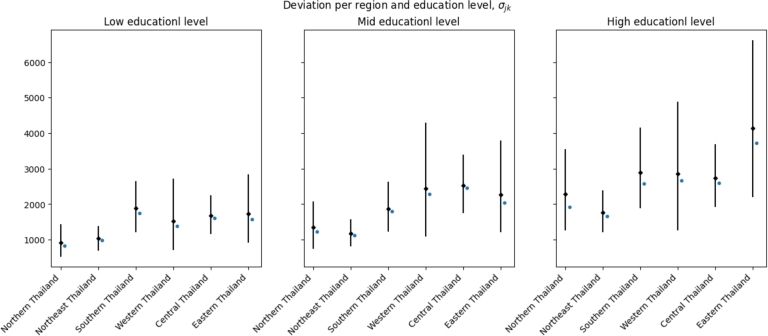
Deviation within-region of the monthly income per region and education level.

**Figure 32:**
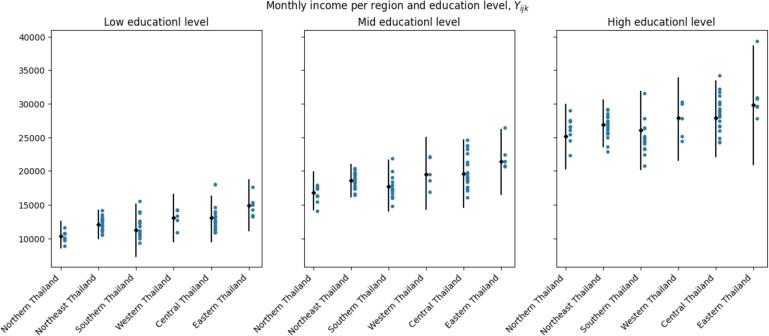
Province average monthly income per region and education level.

**Figure 33:**
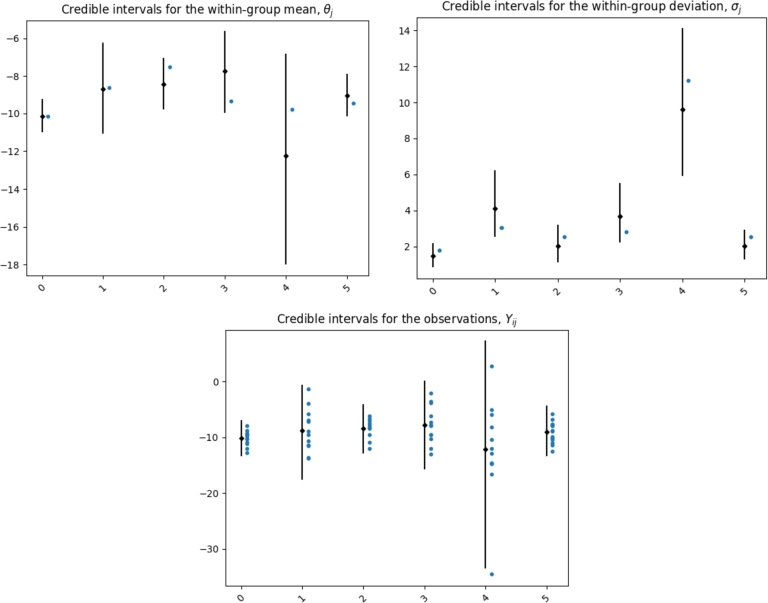
Results considering independent separate models for each group.

**Figure 34:**
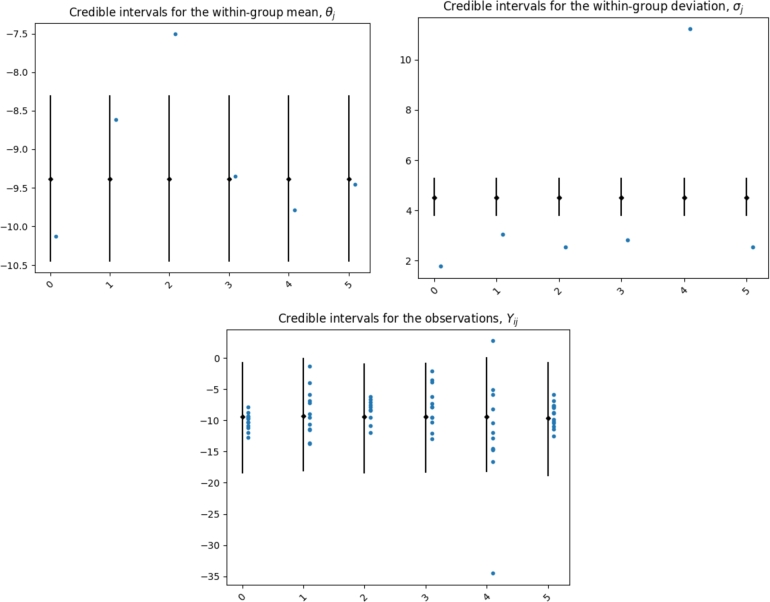
Results considering the complete pooling model for all the groups.

**Figure 35:**
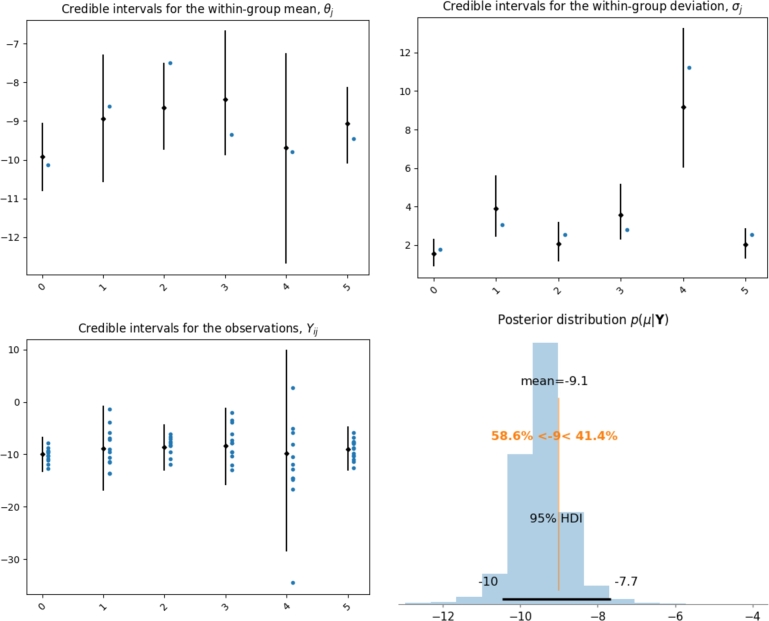
Results considering a hierarchical model. We allow, both the within-group mean and the within-group variance $\sigma_{j}^{2}$ to vary. We considered the prior $\sigma_{j}^{2}|\nu ,\rho^{2}∼\text{Inverse-}\chi^{2}(\nu ,\rho^{2})$. For *ρ*^2^ we used the vague prior , while for *ν* we consider the prior .

**Figure 36:**
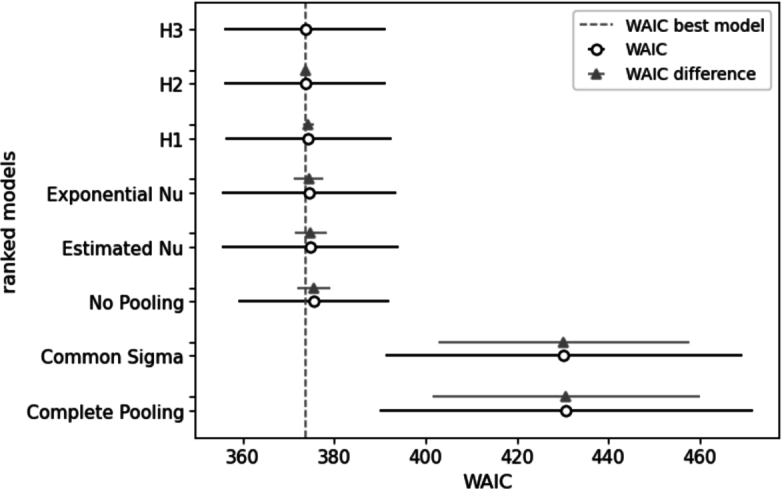
WAIC for the different models.

**Table 7:** Real and estimated values for *ν* and *ρ*.

	Real value	Estimated value
*ν*	4	4.81
*ρ*	3	3.34

**Figure 37:**
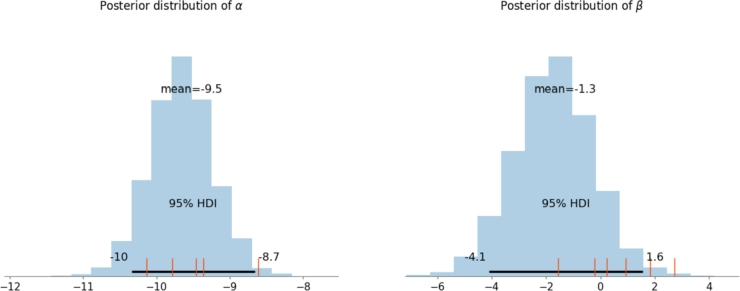
Posterior distribution of *α* and *β* when a unique common model is adjusted to all the data.

**Figure 38:**
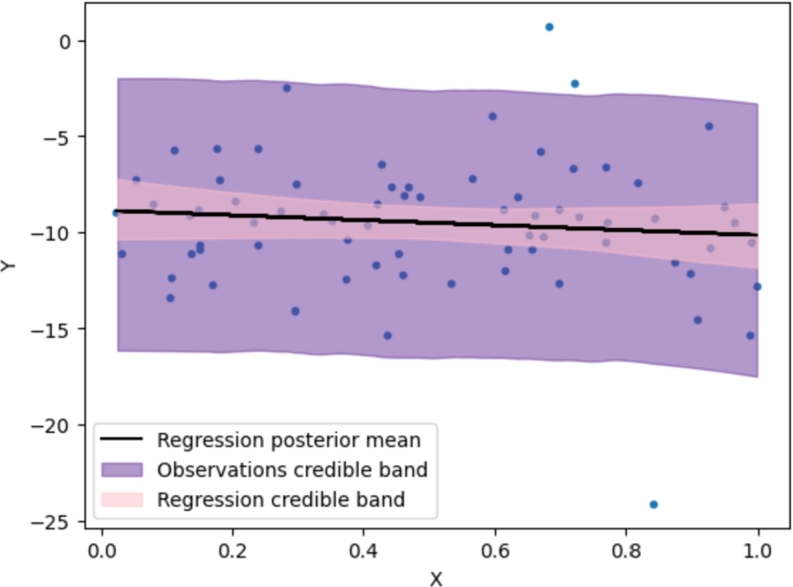
Common regression model.

**Figure 39:**
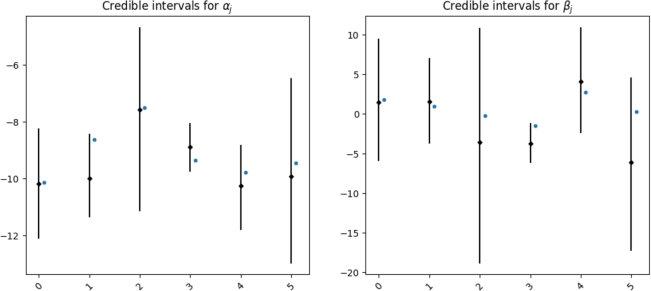
Credible intervals for each *α*_*j*_ and *β*_*j*_ when separate independent models are fitted for each group.

**Figure 40:**
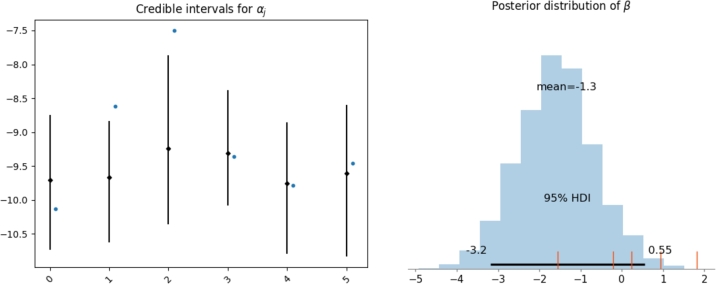
Credible intervals for each *α*_*j*_ and posterior distribution of *β* considering the hierarchical model presented in Section 5.2 where the intercepts of the groups shared a common structure, but the slope is kept the same for all the groups.

**Figure 41:**
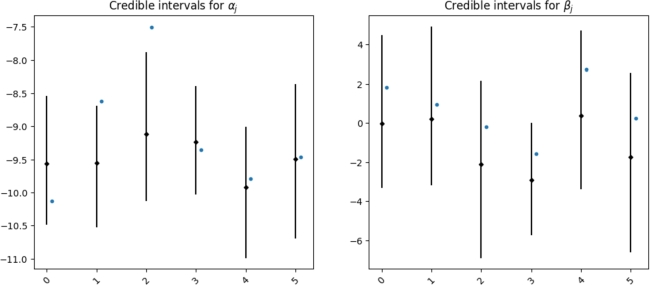
Credible intervals for each *α*_*j*_ and *β*_*j*_ considering the hierarchical model presented in Section 5.3 where the intercept and slope vary between the groups, but they share a common structure.

**Figure 42:**
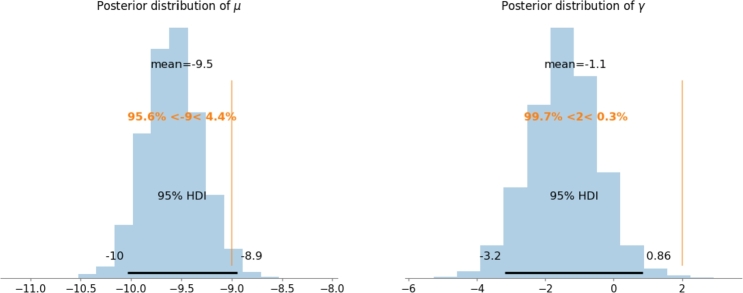
Posterior distributions of *μ* and *γ* considering the hierarchical model presented in Section 5.3.

**Figure 43:**
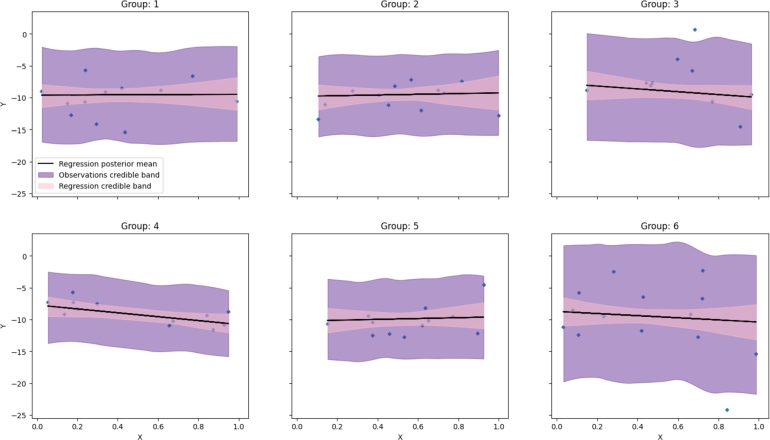
Bayesian hierarchical regression model.

**Figure 44:**
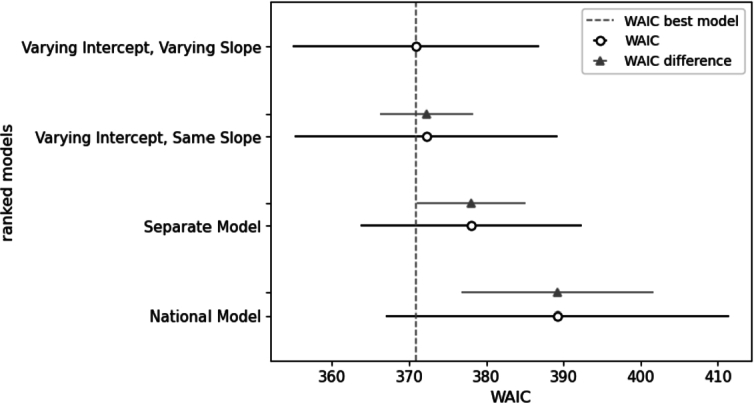
WAIC for the Bayesian hierarchical regression models.

**Figure 45:**
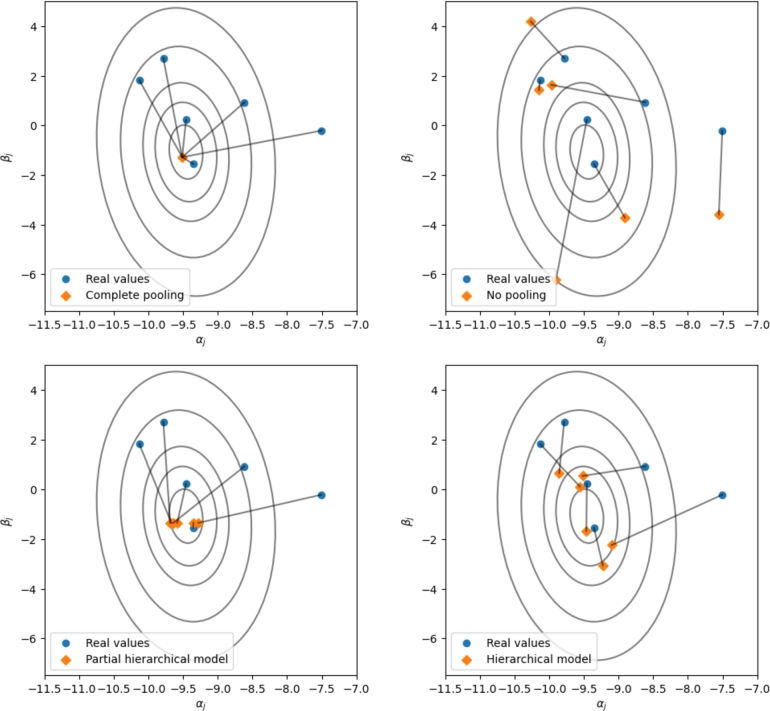
Joint posterior of the slopes and intercepts. The three pictures present each pair (*α*_*j*_,*β*_*j*_) and compare them with the estimates from our different models. Upper left: Comparison with the posterior mean of the pair (*α*,*β*) for the common regression model. Upper right: Comparison with the estimators for separate independent models for each region. Lower left: Comparison with the estimator of each.

## Appendix A Technical appendix

### A.1 WAIC

We can compare the average log-probability for each model to get an estimate of the relative distance of each model from the real distribution of the data. The Bayesian version of the log-probability score is called the log-pointwise-predictive-density (lppd) defined as

$$\text{lppd}=\sum_{i=1}^{n}\log\left[\frac{1}{S}\sum_{s=1}^{S}p\left(Y_{i}|\Theta^{\left(s\right)}\right)\right],$$

where *S* is the number of simulated samples from the posterior distributions, $\Theta^{\left(s\right)}$ is the *s*-th set of sampled parameter values and $p\left(Y_{i}|\Theta^{\left(s\right)}\right)$ is the density of $Y_{i}$ given the parameters $\Theta^{\left(s\right)}$.

However, we should also consider the ‘complexity’ of the model, this complexity is given by the effective number of parameters of the model, labeled $p_{\text{WAIC}}$ and defined as

$$p_{\text{WAIC}}=\sum_{i=1}^{n}V_{\Theta ∼p(\Theta |Y)}\log p(Y_{i}|\Theta ).$$

Because we count with a sample of the posterior distribution of the parameters, this quantity can be well-approximated taking the sample variance of the log-density for each observation *i*, and then summing up these variances.

Therefore, to get a quantitative way to compare the models, we consider the Watanabe-Akaike information criterion (WAIC) defined as

$$\text{WAIC}=-2\text{lppd}+2p_{\text{WAIC}}.$$

In Watanabe's original definition, WAIC is the negative of the average lppd, thus is divided by *n* and does not have the factor 2; it is common to scale it to be comparable with AIC and other measures of deviance.

### A.2 Qualitative characteristics of the parameters and models

Consider the model presented in Section 3.2, and the prior , it is not difficult to prove [42] that

$$\mu |\tau ,\sigma^{2},Y∼N(\overset{ˆ}{\mu },V_{\mu }),$$

where

$$\overset{ˆ}{\mu }=\frac{\sum_{j=1}^{J}\frac{{\overset{¯}{Y}}_{\cdot j}}{{\overset{¯}{\sigma }}_{j}^{2}+\tau^{2}}}{\sum_{j=1}^{J}\frac{1}{{\overset{¯}{\sigma }}_{j}^{2}+\tau^{2}}},\;V_{\mu }=\frac{1}{\sum_{j=1}^{J}\frac{1}{{\overset{¯}{\sigma }}_{j}^{2}+\tau^{2}}}\text{, and}\;{\overset{¯}{\sigma }}_{j}^{2}=\frac{\sigma^{2}}{n_{j}}.$$

Note that

$$\overset{ˆ}{\mu }\underset{\tau \to 0}{\to }\frac{\sum_{j=1}^{J}\frac{{\overset{¯}{Y}}_{\cdot j}}{{\overset{¯}{\sigma }}_{j}^{2}}}{\sum_{j=1}^{J}\frac{1}{{\overset{¯}{\sigma }}_{j}^{2}}}\equiv {\overset{¯}{Y}}_{\cdot \cdot }\text{ and}\;V_{\mu }\underset{\tau \to 0}{\to }\frac{1}{\sum_{j=1}^{J}\frac{1}{{\overset{¯}{\sigma }}_{j}^{2}}}\equiv \varphi^{2}.$$

Using these expressions and Equation (1), we can calculate

(A.1) $$E(\theta_{j}|\tau ,\sigma^{2},Y)=E_{\mu }[E(\theta_{j}|\mu ,\tau^{2},\sigma^{2},Y)|\tau ,\sigma^{2},Y]=E_{\mu }[{\overset{ˆ}{\theta }}_{j}|\tau ,\sigma^{2},Y]=E_{\mu }\left[\frac{\frac{1}{{\overset{¯}{\sigma }}_{j}^{2}}{\overset{¯}{Y}}_{\cdot j}+\frac{1}{\tau^{2}}\mu }{\frac{1}{{\overset{¯}{\sigma }}_{j}^{2}}+\frac{1}{\tau^{2}}}|\tau ,\sigma^{2},Y\right]=\frac{\frac{1}{{\overset{¯}{\sigma }}_{j}^{2}}{\overset{¯}{Y}}_{\cdot j}+\frac{1}{\tau^{2}}\overset{ˆ}{\mu }}{\frac{1}{{\overset{¯}{\sigma }}_{j}^{2}}+\frac{1}{\tau^{2}}},$$

and

$$V(\theta_{j}|\tau ,\sigma^{2},Y)=E_{\mu }[V(\theta_{j}|\mu ,\tau^{2},\sigma^{2},Y)|\tau ,\sigma^{2},Y]+V_{\mu }[E(\theta_{j}|\mu ,\tau^{2},\sigma^{2},Y)|\tau ,\sigma^{2},Y]=E_{\mu }[V_{\theta_{j}}|\tau ,\sigma^{2},Y]+V_{\mu }[{\overset{ˆ}{\theta }}_{j}|\tau ,\sigma^{2},Y]=E_{\mu }\left[\frac{1}{\frac{1}{{\overset{¯}{\sigma }}_{j}^{2}}+\frac{1}{\tau^{2}}}|\tau ,\sigma^{2},Y\right]+V_{\mu }\left[\frac{\frac{1}{{\overset{¯}{\sigma }}_{j}^{2}}{\overset{¯}{Y}}_{\cdot j}+\frac{1}{\tau^{2}}\mu }{\frac{1}{{\overset{¯}{\sigma }}_{j}^{2}}+\frac{1}{\tau^{2}}}|\tau ,\sigma^{2},Y\right]=\frac{1}{\frac{1}{{\overset{¯}{\sigma }}_{j}^{2}}+\frac{1}{\tau^{2}}}+\frac{{\left(\frac{1}{\tau^{2}}\right)}^{2}V_{\mu }}{{\left(\frac{1}{{\overset{¯}{\sigma }}_{j}^{2}}+\frac{1}{\tau^{2}}\right)}^{2}}$$

Note that $E(\theta_{j}|\tau ,\sigma^{2},Y)\underset{\tau \to 0}{\to }{\overset{¯}{Y}}_{\cdot \cdot }$, and $V(\theta_{j}|\tau ,\sigma^{2},Y)\underset{\tau \to 0}{\to }\varphi^{2}$. Meanwhile, $E(\theta_{j}|\tau ,\sigma^{2},Y)\underset{\tau \to \infty }{\to }{\overset{¯}{Y}}_{\cdot j}$, and $V(\theta_{j}|\tau ,\sigma^{2},Y)\underset{\tau \to \infty }{\to }{\overset{¯}{\sigma }}_{j}^{2}$. This implies that both the complete pooling and no pooling models can be seen as extreme cases of the hierarchical model, achieved when τ→0 and τ→∞, respectively. Then, the hierarchical model provides a compromise between these two extreme models.

On the other hand, in [42] it is discussed an empirical approach, based on an analysis of variance (ANOVA), to estimate the parameters $\sigma^{2}$ and $\tau^{2}$, which we now present and analyze here.

The mean square within groups $MS_{W}$ is given by

$$MS_{W}=\frac{1}{J(\overset{¯}{n}-1)}\sum_{j=1}^{J}\sum_{i=1}^{n_{j}}{\left(Y_{ij}-{\overset{¯}{Y}}_{\cdot j}\right)}^{2},\text{ where}\;\overset{¯}{n}=\frac{1}{J}\sum_{j=1}^{J}n_{j},$$

and the mean square between groups $MS_{B}$ by

$$MS_{B}=\frac{1}{J-1}\sum_{j=1}^{J}\sum_{i=1}^{n_{j}}{\left({\overset{¯}{Y}}_{\cdot j}-{\overset{¯}{Y}}_{\cdot \cdot }\right)}^{2},\text{ where}\;{\overset{¯}{Y}}_{\cdot \cdot }=\frac{\sum_{j=1}^{J}\frac{n_{j}}{\sigma^{2}}{\overset{¯}{Y}}_{\cdot j}}{\sum_{j=1}^{J}\frac{n_{j}}{\sigma^{2}}}.$$

Then, unbiased estimators for $\sigma^{2}$ and $\tau^{2}$ are given by ${\overset{ˆ}{\sigma }}^{2\text{ANOVA}}=MS_{W}$ and ${\overset{ˆ}{\tau }}^{2\text{ANOVA}}=\frac{MS_{B}-MS_{W}}{\overset{¯}{n}}$.

The same authors prove that

$$p(\tau |\sigma^{2},Y)\propto p(\tau )V_{\mu }^{1/2}\prod_{j=1}^{J}{\left({\overset{¯}{\sigma }}_{j}^{2}+\tau^{2}\right)}^{-1/2}\exp\left\{-\frac{\left({\overset{¯}{Y}}_{\cdot j}-\overset{ˆ}{\mu }\right)}{2({\overset{¯}{\sigma }}_{j}^{2}+\tau^{2})}\right\}.$$

Note that everything multiplying p(τ) approaches a nonzero constant limit as *τ* tends to zero. Thus, the behavior of the posterior density near τ=0 is determined by the prior density. The usual noninformative function  is not integrable for any small interval including τ=0 and yields a nonintegrable posterior density. Meanwhile, the uniform prior distribution  yields a proper posterior density.

In Fig. 24 we present the posterior conditional distribution of *τ* conditional on the empirical estimate of $\sigma^{2}$, that is $p(\tau |{\overset{ˆ}{\sigma }}^{2\text{ANOVA}})$. We also present the mode of this distribution, and an approximate interval of 0.95 probability. Under conditions of regularity a (1−α)×100% probability interval is given by

$$\left\{\tau :\frac{p(\tau |\sigma^{2},Y)}{p({\overset{ˆ}{\tau }}^{\text{MAP}}|\sigma^{2},Y)}\geq \exp\left\{-\frac{q_{\chi_{1}^{2}}^{1-\alpha }}{2}\right\}\right\},$$

where $q_{\chi_{1}^{2}}^{1-\alpha }$ denotes the quantile of probability 1−α of a $\chi^{2}$ distribution with 1 degree of freedom.

In Fig. 25 we present $E(\theta_{j}|\tau ,\sigma^{2},Y)$, calculated in Equation (A.1), as a function of *τ* and setting the value of $\sigma^{2}$ to ${\overset{ˆ}{\sigma }}^{2\text{ANOVA}}$. We plot a vertical dashed line on the value ${\overset{ˆ}{\tau }}^{\text{MAP}}$. We can observe how this estimated value lies between the extreme cases of the complete pooling and no pooling models.

Consider now the model presented in Section 3.3, and remember that

$$\sigma_{j}^{2}|\theta ,\nu ,\rho^{2},Y∼\text{Inverse-}\chi^{2}(\nu_{j},{\overset{ˆ}{\sigma }}_{j}^{2}),$$

where

$$\nu_{j}=\nu +n_{j},\;{\overset{ˆ}{\sigma }}_{j}^{2}=\frac{\nu \rho^{2}+n_{j}v_{j}}{\nu +n_{j}},\text{and }v_{j}=\frac{1}{n_{j}}\sum_{i=1}^{n_{j}}{\left(Y_{ij}-\theta_{j}\right)}^{2}.$$

Then,

$$E(\sigma_{j}^{2}|\theta ,\nu ,\rho^{2},Y)=\frac{\nu_{j}}{\nu_{j}-2}{\overset{ˆ}{\sigma }}_{j}^{2}=\frac{\nu \rho^{2}+n_{j}v_{j}}{\nu +n_{j}-2},$$

and

$$V(\sigma_{j}^{2}|\theta ,\nu ,\rho^{2},Y)=\frac{2\nu_{j}^{2}}{{\left(\nu_{j}-2\right)}^{2}(\nu_{j}-4)}{\overset{ˆ}{\sigma }}_{j}^{4}=\frac{2{\left(\nu \rho^{2}+n_{j}v_{j}\right)}^{2}}{{\left(\nu +n_{j}-2\right)}^{2}(\nu +n_{j}-4)},$$

from these expressions is easy to see that $E(\sigma_{j}^{2}|\theta ,\nu ,\rho^{2},Y)\underset{\nu \to \infty }{\to }\rho^{2}$ and $V(\sigma_{j}^{2}|\theta ,\nu ,\rho^{2},Y)\underset{\nu \to \infty }{\to }0$. On the other hand, if ν→0, then $\sigma_{j}^{2}|\theta ,\nu ,\rho^{2},Y∼\text{Inverse-}\chi^{2}(n_{j},v_{j})$, corresponding with the no pooling inference.

In Fig. 26 we present $\sqrt{E(\sigma_{j}^{2}|\theta ,\nu ,\rho^{2},Y)}$, as a function of *ν* and setting the value of $\rho^{2}$ to the estimator ${\overset{ˆ}{\rho }}^{2}$ calculated with Equation (3), and substituting $v_{j}$ for the observed variance. We plot a vertical dashed line on the value $\overset{ˆ}{\nu }$ (see Equation (2)). Once again, we can observe how this estimated value lies between the extreme cases of complete pooling and no pooling models.

In Fig. 27 we present a diagram representing the different models considered in Section 3 and their relation with the parameters. On the bottom left of the diagram we present the complete pooling model, which assumes the same within variance for all the regions and the same mean. While on the other extreme of the diagram we present the no pooling model, where separate independent models are adjusted for each region. The hierarchical models are between these two extreme cases. When we consider , we observed through simulations that large values of *h* tend to generate within-region variances similar to the no pooling model, while a common within-region variance is obtained when h→0. Somewhere around these models are our other two approaches, set *ν* to a fixed estimated value $\overset{ˆ}{\nu }$, or assign a prior exponential distribution. Next to each model we present its corresponding WAIC value for the monthly income variable, showing in bold the model with the lowest WAIC (see Table 1).

### A.3 Proofs

#### A.3.1 Conditional posterior distribution of $\rho^{2}$

To calculate the conditional posterior distribution of $\rho^{2}$, note that

$$p(\rho^{2}|\sigma^{2},\nu )\propto p(\rho^{2}|\nu )p(\sigma^{2}|\nu ,\rho^{2})\propto p(\rho^{2})\prod_{j=1}^{J}{\left(\rho^{2}\right)}^{\nu /2}\exp\left\{-\frac{\nu }{2\sigma_{j}^{2}}\rho^{2}\right\}=p(\rho^{2}){\left(\rho^{2}\right)}^{\frac{J\nu }{2}}\exp\left\{-\left(\frac{\nu }{2}\sum_{j=1}^{J}\frac{1}{\sigma_{j}^{2}}\right)\rho^{2}\right\}.$$

Setting  yields

It is immediate from the previous expression that

$$\rho^{2}|\sigma^{2},\nu ∼\text{Gamma}\left(\frac{J\nu }{2},\frac{J\nu }{2{\overset{ˆ}{\rho }}^{2}}\right),$$

where

$${\overset{ˆ}{\rho }}^{2}=\frac{J}{\sum_{j=1}^{J}\frac{1}{\sigma_{j}^{2}}}.$$

Note that $E(\rho^{2}|\sigma^{2},\nu )={\overset{ˆ}{\rho }}^{2}$ is the harmonic mean of the within-groups variances. Thus $\rho^{2}$ models the “common” within-variance.

#### A.3.2 Estimating $\rho^{2}$ and *ν*

Because $\sigma_{j}^{2}∼\text{Inverse-}\chi^{2}(\nu ,\rho^{2})$, then

$$E(\sigma_{j}^{2}|\nu ,\rho^{2})=\frac{\nu }{\nu -2}\rho^{2}$$

and

$$V(\sigma_{j}^{2}|\nu ,\rho^{2})=\frac{2\nu^{2}}{{\left(\nu -2\right)}^{2}(\nu -4)}\rho^{4}.$$

Let be $E_{s^{2}}$ the average of the observed sample within-group variances $s_{1}^{2},\ldots ,s_{J}^{2}$ and $V_{s^{2}}$ their variance. Then, using the method of moments we have

$$E_{s^{2}}=\frac{\overset{ˆ}{\nu }}{\overset{ˆ}{\nu }-2}{\overset{ˆ}{\rho }}^{2}\Rightarrow {\overset{ˆ}{\rho }}^{2}=\frac{\overset{ˆ}{\nu }-2}{\overset{ˆ}{\nu }}E_{s^{2}},$$

and

thus

$${\overset{ˆ}{\rho }}^{2}=\left(1-\frac{2V_{s^{2}}}{2{\left(E_{s^{2}}\right)}^{2}+4V_{s^{2}}}\right)E_{s^{2}}=\left(\frac{2{\left(E_{s^{2}}\right)}^{2}+2V_{s^{2}}}{2{\left(E_{s^{2}}\right)}^{2}+4V_{s^{2}}}\right)E_{s^{2}}.$$

## Appendix B Supplementary figures

### B.1 Income per region and education level

Remember that the expected monthly income for the region *j* and the education level *k* is given by $\theta_{j}+\lambda_{k}$, where $\theta_{j}|\mu ,\tau^{2}∼N(\mu ,\tau^{2})$. Then, fixing the education level, and taking the average over all the regions, i.e. computing the expected value over $\theta_{j}$, we obtain the expected value of the monthly income for the education level *k*, given by $\mu +\lambda_{k}$. We present in Fig. 28 the expected monthly income for each education level. Similarly, Fig. 29 shows the difference of income between the education levels. We can conclude from these Figures that the education level has an important impact on the income of households, when averaging over all the regions, a higher education level is related with a higher income.

We present in Figure 30, Figure 31, Figure 32 our ubiquitous graphs showing the regional average, the regional deviation and the province average of the monthly income. This time, however, we present these quantities for each education level. We observe that the education level has an important impact on income regardless of the region.

## Appendix C Simulation study

### C.1 Hierarchical models with one cluster

For the first part of the simulation study we consider six groups, and simulate data with the next distributions

$$Y_{ij}|\theta_{j},\sigma_{j}^{2}∼N(\theta_{j},\sigma_{j}^{2})\theta_{j}|\mu ,\tau^{2}∼N(\mu ,\tau^{2})\sigma_{j}^{2}|\nu ,\rho^{2}∼\text{Inverse-}\chi^{2}(\nu ,\rho )$$

setting μ=−9, τ=1, ρ=3 and ν=4 where *j* represents the index of group and *i* represents the index of observation. While the number of observations for each group is simulated from independent shifted Poisson distributions having support in {10,11,…} and with rate parameter equal to three.

Most of the observations made through the main part of this article remains for the simulated data. Thus, we limit ourselves here to show analogous graphs to those presented in the main part of the article, adding minor comments when we consider them necessary.

As in the main part of the present article, we start our simulation study considering independent models for each group. Fig. 33 shows our usual credible intervals for each $\theta_{j}$, $\sigma_{j}$ and the observations within each group $Y_{ij}$, with the only difference that now next to the credible intervals we show the real simulated values.

As commented in the main part of the article, we observe wide intervals, overlapping in most of their values, which indicates that a complete pooling model might be more appropriate for the data. In Fig. 34 we show the analogous results when a complete pooling model is considered. We observe a poor performance for this model when tries to infer the real mean and standard deviation of each group.

Due to the poor performance of the complete pooling model and the large variance when separate models are implemented, we present the hierarchical model as a trade-off that might reduce the uncertainty of the estimations while keeping a good performance to explain the data. For brevity, we only show in Fig. 35 the analogous results of Fig. 7 for the hierarchical model with the lowest WAIC which, for the simulated data, corresponds with the case where . Reminding that a model with lower WAIC performs better than a model with higher WAIC.

In Fig. 36 we present the WAIC comparison of the different models. We can observe that for these simulated data, except for the complete pooling model and the hierarchical model with common within-groups deviation *σ*, most of the models present similar values for the WAIC.

Finally, in Table 7 we compare the real values of *ν* and *ρ* against their estimated values given by Equations (2) and (3), where we can observe that these quantities can be properly estimated by the proposed procedure.

### C.2 Bayesian hierarchical regression

The second part of our simulation study is devoted to Bayesian hierarchical regression. For this part, we keep six different groups, and simulate data according to the following distributions

$$Y_{ij}|\alpha_{j},\beta_{j},\sigma_{j}∼N(\alpha_{j}+\beta_{j}(X_{ij}-{\overset{¯}{X}}_{\cdot j}),\sigma_{j}^{2})\alpha_{j},\beta_{j}|\mu ,\tau^{2},\gamma ,\zeta^{2}∼MVN\left(\left[\begin{array}{c} \mu \\ \gamma \end{array}\right],\left[\begin{array}{cc} \tau^{2} & 0\\ 0 & \zeta^{2} \end{array}\right]\right)\sigma_{j}^{2}|\nu ,\rho^{2}∼\text{Inverse-}\chi^{2}(\nu ,\rho^{2})$$

setting μ=−9, τ=1, γ=2, ζ=3, ρ=3 and ν=4. While the number of observations for each group is simulated from independent shifted Poisson distributions having support in {10,11,…} and with rate parameter equal to three.

In Fig. 37 we present the posterior distribution of *α* and *β* when a common model is adjusted for all the data, we have added the real values of each $\alpha_{j}$ and $\beta_{j}$. Fig. 38 presents a scatter plot of the simulated data adding the fitted model to the graph. Analogously to Fig. 37, Figure 39, Figure 40, Figure 41 show the posterior distribution or credible intervals for the intercept and slope of the regression models considered in Section 5. We observe from these figures that, considering a single common slope for all the groups, it tends to underestimate the slope of some groups. In Fig. 42 we present the analogous result presented Fig. 20, which shows the posterior distributions of *μ* and *γ* for the Bayesian hierarchical regression model, allowing the intercepts and the slopes to share a common structure between groups. We observe that, for these data, both *μ* and *γ* tend to be underestimated.

In Fig. 43 we present the fitted model which introduces a hierarchical structure of the intercepts and slopes of the groups. In Fig. 44 we show the WAIC of the considered regression models, where the Bayesian hierarchical model presents the lowest WAIC between all the models, while a single common regression model presents the largest WAIC. Finally, in Fig. 45, we present the joint distribution for the intercepts and slopes, comparing their real simulated values against their estimated values for the four models considered in this article.

## Footnotes

1 $N(\theta ,\sigma^{2})$ denotes a normal distribution with location *θ* and scale *σ*.

2 denotes the indicator function, defined as

for some set *A* where *x* is properly defined.

3 Inverse-$\chi^{2}(\nu ,\sigma^{2})$ denotes a scaled inverse $\chi^{2}$ distribution with *ν* degrees of freedom and scale $\sigma^{2}$.

4 Gamma(α,β) denotes a gamma distribution with shape parameter *α* and rate parameter *β*.

5 Source for the raw map of Thailand: https://github.com/cvibhagool/thailand-map.
